# Emperors, admirals and giants, zebras, tigers and woolly bears: casting a broader net in exploring heparin effects on Lepidoptera wing patterns

**DOI:** 10.12688/f1000research.16926.3

**Published:** 2020-12-04

**Authors:** Andrei Sourakov

**Affiliations:** 1 McGuire Center for Lepidoptera and Biodiversity, Florida Museum of Natural History, University of Florida, Gainesville, Florida, 32611, USA

**Keywords:** wing pattern, butterflies, moths, sulfated polysaccharides, evolution of development

## Abstract

**Background:** Studies of heparin effects on Lepidoptera wing patterns have been restricted to a small number of species. I report observations from experiments on a broader range of taxa, including first results from swallowtails, tiger moths and microlepidoptera.

**Methods:** Heparin injections were made in prepupae and pupae of *Junonia coenia *(common buckeyes), *Agraulis vanillae *(gulf fritillaries), *Heliconius charithonia *(zebra longwings), *Asterocampa clyton *(tawny emperors) *, Danaus plexippus *(monarchs), *Vanessa atalanta *(red admirals); *Heraclides cresphontes *(giant swallowtails), *Pterourus troilus *(spicebush swallowtails), *Protographium marcellus *(zebra swallowtails), *Battus polydamas *(polydamas swallowtails); *Hypercompe scribonia *(giant leopard moths), *Estigmene acrea *(acrea moths), *Hyphantria cunea *(fall webworm moths) *, Utetheisa ornatrix* (ornate bella moths); *Glyphodes sibillalis* (mulberry leaftier).

**Results:** Heparin sometimes altered the entire pattern in a dramatic way, sometimes caused changes locally. ****In buckeyes, the previous heparin study conducted on pupae was compared to injections made at a prepupal stage. In gulf fritillaries, zebra longwings and tawny emperors, the dramatic changes occurred throughout their wings, while in monarchs, changes were restricted to wing margins. Changes achieved in red admirals, show that heparin action is unrelated to the original color. In swallowtails, transformations were restricted to border system, indicating higher levels of stability and compartmentalization of wing patterns. In mulberry leaftier, changes were restricted to the marginal bands. In tiger moths, elongation of black markings led to merging of spots; in the ornate bella moth, it was accompanied by an expansion of the surrounding white bands, and results were compared to the effects of colder temperatures.

**Conclusions: **Using pharmaceutical intervention demonstrates that there are many similarities and some very significant differences in the ways wing patterns are formed in different Lepidoptera lineages. By creating a range of variation one can demonstrate how one pattern can easily evolve into another, aiding in understanding of speciation and adaptation processes.

## Introduction

The wing patterns of butterflies and moths are not only physical characteristics that interact with their surroundings according to the laws of physics, such as through the absorption or reflection of heat; the spectacular array and the subtle variations in wing patterns found among ca. 160,000 Lepidoptera species are due to the fact that these patterns are also ornaments, and as such, are there to interact with an observer—whoever that observer may be—contributing not only to natural but also to sexual selection. Hence, understanding how these ornaments evolve and how they are regulated during their development would help understand the process of selection.

There is by now ample evidence that heparin-like glycosaminoglycans are involved in wingless signaling and that adding them at the right stage of insect development can increase the activity of *Wnt*-ligand products (e.g., Binari *et al.*, 1997). Since pioneering experiments on the common buckeye, it has been known that the wing pattern of a butterfly can be altered by heparin injection during the pupal stage ( Serfas & Carroll, 2005). Heparin is a sulfated polysaccharide, whose action was demonstrated to increase diffusion in the intracellular space and activity of the *Wnt*-family ligand gene products hence distorting, and sometimes destroying, individual pattern elements ( Fuerer & Nusse, 2010). According to the latest work ( Fenner *et al.*, 2020), *Wnt* genes are highly conserved throughout Lepidoptera, but their deployment seems to be highly divergent among different lineages. For instance, a dogface butterfly of the family Pieridae deploys a different set of *Wnt* genes than were found to be deployed by the Nymphalidae, which heretofore have been the focal point of wing pattern evo-devo research. Of course, it is very important to note that, while our knowledge about wing-pattern development is multiplying daily, we are still just scratching the surface of the vast diversity of Lepidoptera lineages and wing patterns. In the present publication, I am attempting to introduce additional species from different lineages into the equation in the hope of contributing to the overall understanding of wing pattern homologies. These species are common and easy to rear, so all of them are potential candidates for a more in-depth approach using gene expression and CRISPR techniques.

This test of injecting heparin into a developing Lepidoptera, sometimes is interpreted as having an opposite effect to the loss-of-function test by *WntA* knockout (using CRISPR technology), as it seemingly leads to an expansion of Wnt-positive wing pattern elements (e.g., Martin & Reed, 2014). The *WntA* knockout test now has been used on a number of nymphalid butterflies by several butterfly wing pattern research groups and provides an excellent point of reference for the present study. For example, it was recently successfully used to demonstrate how co-mimics within the genus *Heliconius* employ different regulatory pathways to achieve similar patterns ( Concha *et al.*, 2019).

It is important to note at this point that, while we do understand to some extent the influence of heparin on wing pattern formation, our understanding is limited. For instance, Ding *et al.* (2019) demonstrate that there are eight different Wnt-family genes that are active in Lepidoptera during different stages of development and in different cells. Which of them are affected when and how by heparin may be greatly complicated by the fact that our knowledge of gene expression is normally based on a few model species (e.g., silk worm, *Bombyx mori*) and may not be universally applicable throughout Lepidoptera. In the recent wing pattern study, a first for pierid butterflies, Fenner *et al.* (2020) demonstrated that, while *Wnt* genes involved in pattern formation are different from those of Nymphalidae and the prime target thought to be affected by heparin in the nymphalids, the *WntA*, was not expressed, the heparin test can still affect pattern development, albeit in a sex-limited manner. Nevertheless, if the “heparin test” is applied to, for example, eyespot-bearing species from the same family, where a complex eyespot consisting of several concentrically organized circles are positioned identically on the wing, and the eyespots respond to heparin in different ways in different species, it is prudent to hypothesize that these eyespots, while possibly having a common origin, are now under different developmental controls. Such a test was recently applied to the saturniid moths (io and polyphemus moths) and they demonstrated marked differences in responses ( Sourakov & Shirai, 2020).

When, in 2017, I injected heparin into two prepupae, along with several pupae, of the io moth, it was a deviation from standard practice, which called for targeting early pupal stages. In that experiment, injections into prepupae and pupae achieved similar pattern transformations ( Sourakov, 2017). The common buckeye, *Junonia coenia*—a nymphalid species for which heparin-induced wing pattern manipulation in pupae has already thoroughly been explored by Serfas & Carroll (2005) —offered a good model for examining whether injection at the prepupal stage resulted in different transformations. Additionally, I present here the results of experiments conducted on the following Lepidoptera species: Nymphalidae: the red admiral ( *Vanessa atalanta*); the monarchs ( *Danaus plexippus*), the gulf fritillaries ( *Agraulis vanillae*), the zebra longwings ( *Heliconius charithonia*), the tawny emperors ( *Asterocampa clyton*); Papilionidae: the giant swallowtail ( *Heraclides cresphontes*), the spicebush swallowtail ** ( *Pterourus troilus*), the zebra swallowtail ( *Protographium marcellus*), and the polydamas swallowtail ( *Battus polydamas*); Erebidae: the leopard moth ( *Hypercompe scribonia*), the acrea moth ** ( *Estigmene acrea*), the fall webworm ( *Hyphantria cunea*) *,* the ornate bella moth ( *Utetheisa ornatrix*); and Crambidae: the mulberry leaftier ( *Glyphodes sibillalis*).

All of these species are common to Florida. Collectively, they offer a good basis for comparison as they represent several different Lepidoptera (see Table 1 for subfamilies/tribes they represent) lineages, for which we now have a good time-referenced phylogeny ( Kawahara *et al.*, 2019).

**Table 1.** Variation in survival after heparin injection, by species and developmental stage at time of injection.

Species	Taxonomy	Survival by stage of injection		
		Prepupa	Pupa	Total
Common buckeye, *Junonia coenia*	Nymphalidae: Nymphalinae	64% (N=55)	0% (N=16)	49% (N=71)
Red admiral, *Vanessa atalanta*		n/a	100% (N=2)	100% (N=2)
Gulf fritillary, *Agraulis vanillae*	Nymphalidae: Heliconiinae	70% (N=31)	73% (N=11)	70% (N=42)
Zebra longwing, *Heliconius charithonia*		0% (N=1)	100% (N=14)	93% (N=15)
Tawny emperor, *Asterocampa clyton*	Nymphalidae: Apaturinae	20% (N=10)	21% (N=14)	21% (N=24)
Monarch, *Danaus plexippus,* trial 1	Nymphalidae: Danainae	75% (N=8)	42% (N=24, 1-3ul) 73% if 1ul (N=11)*	50% (N=32)
Monarch, *Danaus plexippus*, trial 2		100% (N=1)	92% (n=50)	92% (N=51)
Giant swallowtail, *Heraclides cresphontes*	Papilionidae: Papilionini	42% (N=26)	26% (N=34)	33% (N=60)
Spicebush swallowtail, *Pterourus troilus*		0% (N=1)	40% (N=5)	33% (N=6)
Zebra swallowtail, *Protographium marcellus*	Papilionidae: Leptocirini	0% (N=5)	0% (N=14)	0% (N=17)
Polydamas swallowtail, *Battus polydamas*	Papilionidae: Troidini	50% (N=2)	95% (N=20)	91% (N=22)
Mulberry leaftier, *Glyphodes sibillalis*	Crambidae	17% (N=6)	17% (N=6)	17% (N=12)
Acrea moth, *Estigmene acrea*	Erebidae: Arctiina	100% (N=5)	92% (N=12)	94% (N=17)
Leopard moth, *Hypercompe scribonia*		78% (N=18)	75% (N=8)	77% (N=26)
Bella moth, *Utetheisa ornatrix*, brood 1	Erebidae: Calliomorphina	64% (N=36)	92% (N=25)	77% (61)

## Methods

### Caterpillar collection and rearing

Several hundred caterpillars of all species used in this study were collected in Gainesville, Florida or obtained from eggs laid by a wild-caught female. They were then raised to pupation, with over 500 of them used as experimental groups (see Table 1 for numbers per species). When practical, unmanipulated and H _2_O-injected individuals were reared in the same conditions for each species as controls, but in some cases, analysis of specimens in the scientific collection of the McGuire Center for Lepidoptera and Biodiversity (MGCL) was used to understand the scope of natural variation.

Caterpillars were reared inside plastic bags or containers on the foliage of their respective hostplants as follows: on *Plantago lanceolata* (buckeyes); on *Urtica* sp. (red admirals): on *Passiflora incarnata* (gulf fritillaries); on *Celtis laevigata* (tawny emperors); on *Ascelpias curassavica* (monarchs) on *Zanthoxylum clava-herculis* and *Z. fagara* (giant swallowtails); on *Cinnamomum camphora* and *Sassafras albidum* (spicebush swallowtails); on *Aristolochia tagala* (polydamas swallowtail); on *Asimina triloba* (zebra swallowtail); on *Tradescantia ohiensis* and *Melilotus alba* (acrea and leopard moths); on *Crotalaria lanceolata* (ornate bella moth); on *Juglans nigra* (fall webworm); and on *Morus* sp. (mulberry leaftier moth). The buckeyes and the polydamas swallowtails were reared at natural light condition at 27°C; all others, at 24-hour light in the lab at 23°C. The immature stages used to generate the graph in Figure 8 were weighed using Metter Toledo AL104 analytical balances.

### Heparin injections and observations

Heparin injections were made with either a 10-µl Hamilton syringe or a 0.3-ml hypodermic syringe. In the latter case, the amount was measured out with the 10-µl micropipette. The heparin solution was made from porcine-derived heparin sodium salt (manufactured by MP Biomedicals, Inc., supplied by Fisher Scientific Catalogue #194114 (M.W. 3000 g/mol)) dissolved in distilled water. Different concentrations and volumes of heparin solution were injected. Concentrations and volumes used were different for each species (according to their size), and were sometimes varied in order to achieve variation in response ( Table S1) ( Sourakov, 2018c) and Extended Data ( Sourakov, 2020). The rational for how to vary the dosage can only be described by the words “trial and error.” To achieve transformation in the saturniid moths in Sourakov & Shirai (2020), I had to increase dosage dramatically compared to the experiments by others on small butterflies. Because species size varies widely, and it seems wing transformation thresholds do as well, it is good practice to conduct at least two trials: a pilot study that tests a variety of stages and times before (BP) and after (AP) pupation, and then a second study that narrows both the time window and the dosage. Experiments also investigated the effect of temperature on an individual’s response to injection ( Table S1) ( Sourakov, 2018c) and Extended Data ( Sourakov, 2020). When the temperature was lowered from 23°C to 16°C, the experimental animals were placed in this temperature 1–3 hours prior to injection and left there for at least 24 hours afterwards. If a prepupa was injected at this temperature, it was allowed to pupate at 16°C and left an additional 24 hours as pupa at 16°C before being returned to 23°C. For many individuals, the exact time from injection to or after pupation was determined using time-lapse photography with precision of 0.5–1 hour, while for others it was only determined approximately using visual observations ( Table S1) ( Sourakov, 2018c) and Extended Data ( Sourakov, 2020). While heparin dosage varied, so did the volume of injected solution, both of which have independent effects on survival. While the numbers of control individuals varied depending on availability, from 5 (as in the case of mulberry leaftiers) to 53 (as in the case of ornate bella moth) they were also supplemented by consulting the collection holdings of the McGuire Center for Lepidoptera and Biodiversity (MGCL) for these species from the Gainesville area. MGCL is where the voucher specimens resulting from this study are also deposited.

## Results and discussion

### Effect on wing pattern

**Figure 1.**
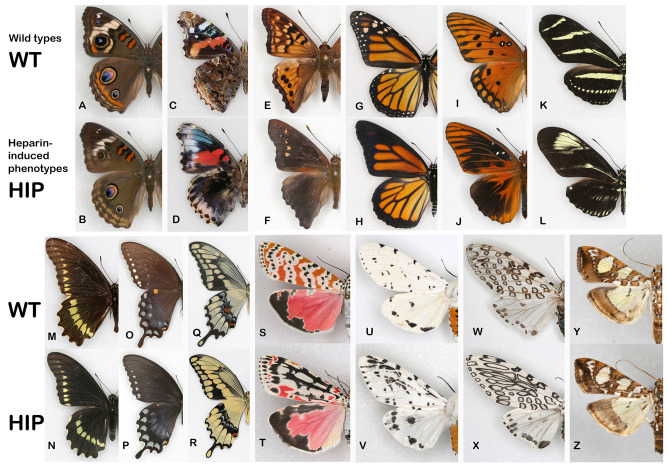
The experiment at a glance: wild types (WT) vs. heparin-induced phenotypes (HIP) in 13 species of Lepidoptera from four families. ( **A**, **B**) The common buckeye ( *Junonia coenia*); ( **C**, **D**) the red admiral ( *Vanessa atalanta*); ( **E**, **F**) the tawny emperor ( *Asterocampa clyton*); ( **G**, **H**) the monarch ( *Danaus plexippus*); ( **I**, **J**) the gulf fritillary ( *Agraulis vanillae*); ( **K**, **L**) the zebra longwing ( *Heliconius charithonia*); ( **M**, **N**) the polydamas swallowtail ( *Battus polydamas*); ( **O**, **P**) the spicebush swallowtail ( *Pterourus troilus*); ( **Q**, **R**) the giant swallowtail ( *Heraclides cresphontes*); ( **S**, **T**) the ornate bella moth ( *Utetheisa ornatrix*); ( **U**, **V**) the leopard moth ( *Hypercompe scribonia*); ( **W**, **X**) the acrea moth ( *Estigmene acrea*); ( **Y**, **Z**) the mulberry leaftier ( *Glyphodes sibillalis*).

***Overall results*.** The transformations were achieved in 13 species of Lepidoptera, nine butterflies and four moths ( Figure 1). Six Nymphalidae butterflies represent four subfamilies: Nymphalinae, Apaturinae, Heliconiinae and Danainae. Three Papilionidae species represent two Tribes: Troidini and Papilionini, and two subgenera, Pterourus and Heraclides are represented within the latter. The three Erebidae moths from what used to be considered a tiger moth family, now known as subfamily Arctiinae, represent two subtribes: Callimorphina and Spilosomina. Finally, a single “micromoth” from the family Crambidae was also tested. As pictured in Figure 1, the transformations achieved are, at first glance, as variable as the patterns themselves, with the only common theme being an expansion-diffusion of marginal elements. However, the common themes can be determined if one considers the “nymphalid groundplan” ( Otaki, 2020; Schwanwitsch, 1924) and “arctiid archetype” ( Gawne & Nijhout, 2020) – two currently existing paradigms of wing pattern homologies in Lepidoptera. Discussions of individual species by family are presented below.

**NYMPHALIDAE**

**Figure 2.**
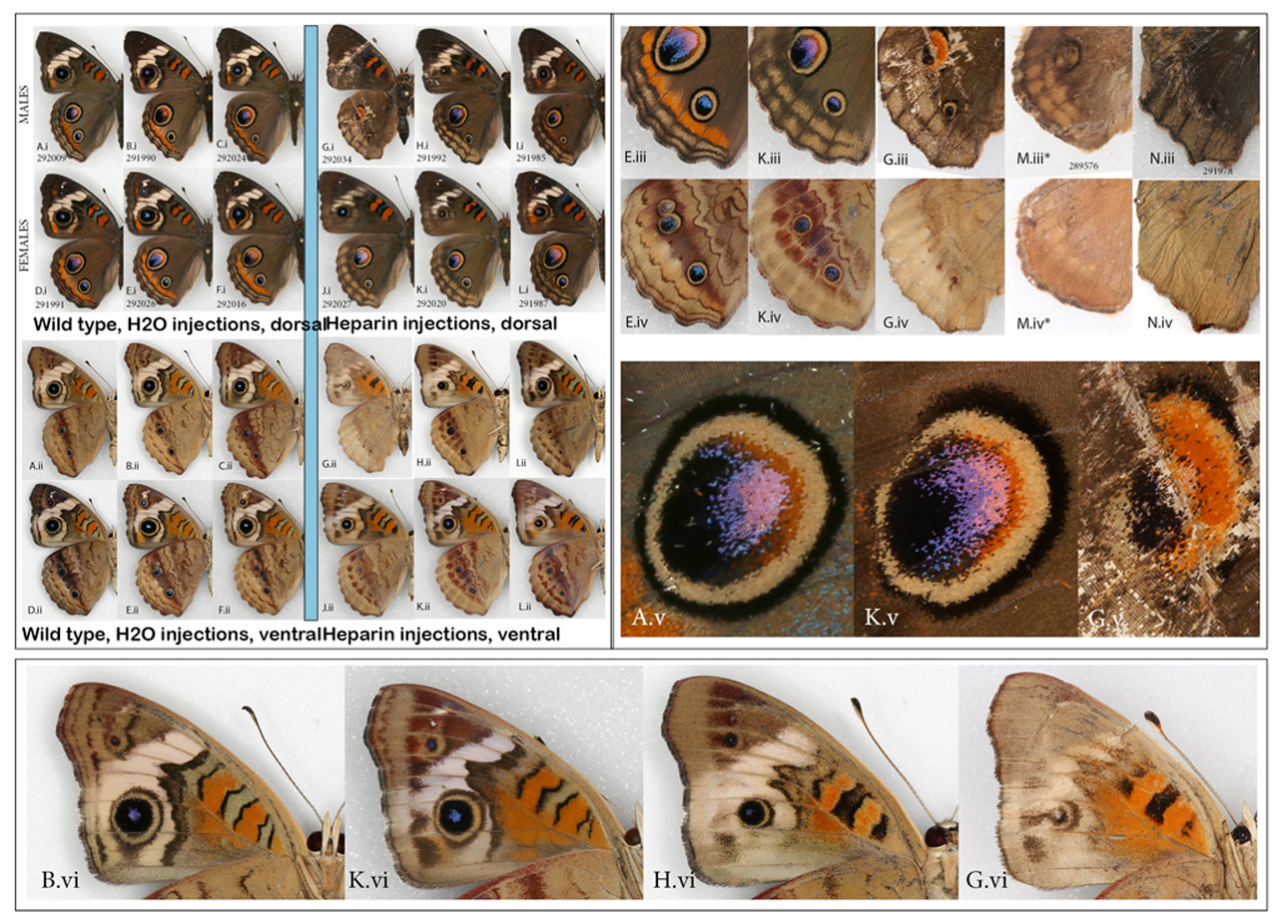
The common buckeye, *Junonia coenia*. Heparin-induced wing pattern changes (right) vs. controls (left) on the dorsal ( **i**) and ventral ( **ii**) surfaces. ( **A**– **F**) Control group: ( **A**– **C**) males and ( **D**– **F**) females injected H _2_O as prepupae. ( **G**– **L**) Experimental group: ( **G**– **I**) Males and ( **J**– **L**) females injected heparin as prepupae. For both groups, specimens shown are the ones in which the dorsal orange hindwing band was expressed the least. ( **iii**) Close-up of HWd, ( **iv**) Close-up of HWv, ( **v**) Close-up of HWd large eyespot, ( **vi**) Close-up of FWv. All showing gradual change as the heparin influence increases. *This individual was dissected out of the pupa, so figured is smaller un-inflated wing at larger relative size compared to the rest. See Extended Data ( Sourakov, 2020) for details.

**NYMPHALINAE:** ***Common buckeye, Junonia coenia, and the red admiral, Vanessa atalanta.*** The buckeye was a species for which Serfas & Carroll (2005) provided excellent baseline information regarding heparin effects on the wing pattern. The present study therefore focused on the prepupal stage to compare the effect of heparin with that resulting from pupal injections (the method used by the aforementioned authors). The heparin-induced changes achieved here consisted of the orange parafocal element being lost as the distal marginal and submarginal bands shift basally and overrun it. The width and clarity of the normally cream-colored markings on the dorsal forewing surface is reduced, along with the reduction of the forewing eyespots. This resulted in specimens that are overall less colorful dorsally ( Figure 2Ai–Li). Ventrally, the same individuals exhibited a reduction in the size of eyespots and loss of definition in some of the wing pattern elements ( Figure 2Aii–Lii). While buckeyes are quite variable in nature, the three “control” groups (non-manipulated, H _2_O-injected, and wild adults collected from the same locality as caterpillars) were similar to each other. The experimental heparin-injected group, on the other hand, ranged from displaying phenotypes that were not found in any of the control groups (as in Figure 2G–N) to presenting normal coloration ( Figure S1) ( Sourakov, 2018c). An almost complete modification of the pattern, as shown in Figure 2G–N, occurred only three times but these individuals did not appear viable, and were unable to climb or fly, although two of them made it out of the pupae and spread their wings.

The present results are mostly similar to those obtained by the Serfas & Carroll (2005) study, which gives confidence in the methods used, however, there are also a few minor differences that make results from the buckeye part of the present study of some interest. While Serfas and Carroll, who experimented on pupae, achieved a gradual reduction in eyespot size with increased heparin dosage, which began once the eyespot integrity was violated (the outer black ring was no longer intact distally), they showed that this effect depends greatly on the timing of the injection. In contrast, in the present study conducted on prepupae, while the threshold at which the eyespots’ integrity is broken (outer black ring is no longer complete) was readily achieved, the dHW eyespot size/color remained largely unchanged (e.g., Figure 2 K.iii). In Serfas & Carroll (2005, p. 418, Figure 2) the dHW eyespots and margins lost their color and definition progressively as the large dose (15 ug) of heparin was injected closer and closer in time to 5 hAP, with minimal to no change past the 20 hAP mark. With the increase of dosage from 0.3 to 30 ug at 5 hAP, Serfas & Carroll observed progressive reduction not only in the color and definition of the dHW eyespots but also in their size, so that at injections of 30 ug, they disappeared almost entirely ( Serfas & Carroll, 2005, p. 418, Figure 1). Their results indicated that past a certain point following pupation, the mapping of the dHW eyespot has been achieved, so the only thing that is continuously affected by heparin is scale color and definition of the rest of the wing pattern. When these authors varied the dosage at 5 hAP, both the layout and the color of the dHW eyespot (as well as the rest of the wing pattern) were still being formed, and heparin affected all elements in a dose-dependent manner. In our experiment, the specimen in Figure 2Gv demonstrates that not all colors within the dHW eyespot are impacted negatively by heparin: the red part expanded instead of contracting. This, combined with the next level of heparin effect shown in Figures 2M.iii, N.iii suggests that it may be the expansion of the margins basally and not the negative effect of heparin on eyespots that leads to eyespot reduction and obliteration.

Serfas & Carroll’s (2005) experiments also demonstrated that different wing surfaces have different timing of pattern mapping, as the vFW behaves differently from the dHW: background color pattern, the definition and size of eyespots all show gradual and continuous dependence on the timing of the injection, with the heparin effect becoming progressively weaker as 15 ug injections move further from the 5 hAP mark towards 20 hAP. In the present study, it is also rather clear that the degrees to which different wing surfaces are affected by heparin are different, with, for example, dHW eyespots persisting longer than the corresponding vHW eyespots and with vFW eyespots being even more resistant to change ( Figure 2 (iii, iv, v)). Many of the butterflies in the experimental group of buckeyes differ from controls in the color and definition of wing margins. These, seemingly viable, individuals have similarly sized dHW eyespots, but their FW eyespots are reduced on both wing surfaces. On close observation one can see that the outer ring of the dHW eyespots is “broken” distally ( Figure 2(v)) and that marginal border system (MBS, term following Otaki, 2017) is greatly diffused ( Figure 2(iii)). A similar transformation of the MBS as well as the melanisation of central symmetry system (CSS) elements can be observed on dFW ( Figure 2(vi)). The type of transformation, exemplified by specimens shown in Figure 2G,M,N is more dramatic: the entire pattern is affected, including change of color and disappearance of eyespots. Sharp reduction of dHW eyespot size is also accompanied by the loss of viability in these individuals and correlates with larger doses of heparin.

There are several possible explanations for why these two distinct modes of transformation, the moderate and the extreme (instead of the gradual time-/dose-dependent changes observed by Serfas & Carroll (2005)) are achieved here: (a) at the prepupal stage, heparin’s effects may be negated by something (e.g., immune system, enzymes or presence/absence of hormones), (b) the dHW eyespot mapping process may be an extended process and once heparin is eliminated from the system, it resumes, (c) heparin’s effect on eyespots is secondary - other pattern elements (e.g. MBS) expanded by the effect of heparin influence what happens to the eyespots. The latter explanation seems to be the one favored by Martin & Reed (2014, p. 373).

If explanations (a) or (b) are correct, similar experiments could provide an excellent opportunity for understanding how the Wnt pathway (if that is indeed what heparin targets) interacts with other physiological processes. If explanation (c) is correct, however, this would mean that eyespots form their own “wing compartment” resistant to change induced by morphogens from the surrounding wing tissue.

The cases in the present study where the entire wing pattern is almost completely erased (as in Figure 2G) must be a result of heparin either being carried through after pupation and affecting eyespots at the time of their development, or (more likely) rendering the entire wing insensitive to signaling during pattern formation because the boundaries of pattern elements are destroyed. Results from additional Lepidoptera species from a variety of lineages presented below support the compartment hypothesis (c). For additional discussion of heparin influence on eyespots, see Sourakov & Shirai (2020).

Analysis of the wing patterns of all surviving individuals injected as prepupae with heparin (N=21) suggests that injection at the early prepupal stage (as defined in Sourakov, 2018c) is more likely to lead to survival and transformation: among 10 experimental individuals sporting the most transformed wing patterns, three were injected as late prepupae and seven as early prepupae.

The taxonomy of the genus *Junonia* in the New World has been complicated by the variability in wing patterns found within and among species and populations. For the widespread species *J. coenia*, this has led to the generation of various taxonomic hypotheses, and modern approaches have recently been used to test them (e.g., Lalonde *et al.*, 2018; Peters & Marcus, 2017). Laboratory-generated aberrations, such as the ones figured in the present study, may be useful for understanding the source of variability found among natural populations and perhaps even help in making taxonomic decisions. In fact, the cryptic taxon *J. grisea* shows, upon closer examination, similar differences from *J. coenia* that are achieved here through heparin injections ( Lalonde & Marcus, 2018). While mt-DNA barcoding is powerless in delineating the species in these and other closely related species of *Junonia*, they are distinguishable by the *wingless* locus ( Borchers & Marcus, 2014; Gemmell *et al.*, 2014 and references above). Hence, *Wnt*-ligand sequences (presumably affected by heparin) could also have been associated with the divergence of these species.

**Figure 3.**
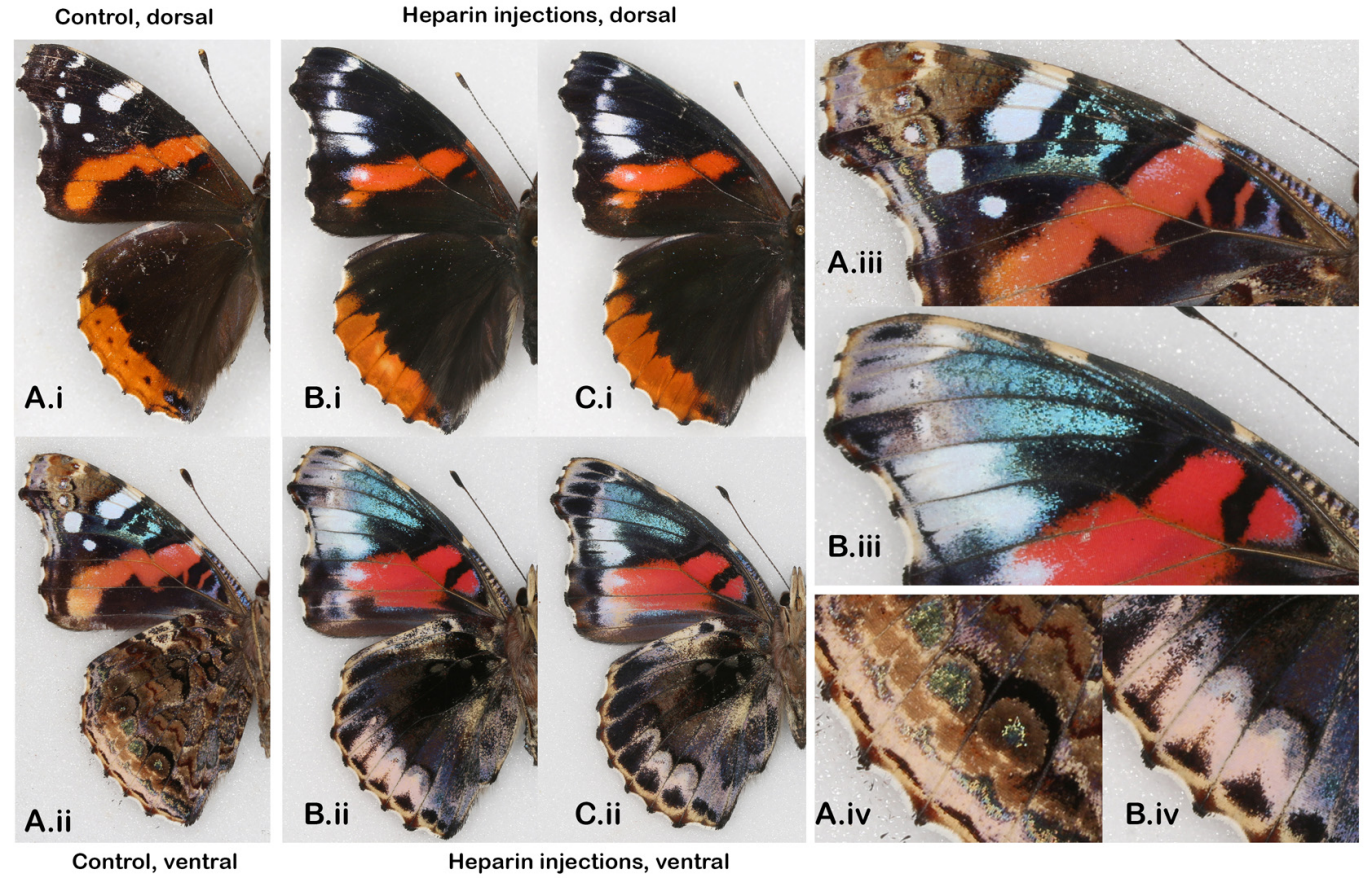
The red admiral, *Vanessa atalanta*. Wild type ( **A**) vs. Heparin-induced wing pattern changes ( **B**– **C**) after injecting heparin into pupae; ( **i**) the dorsal and ( **ii**) ventral surfaces ( **iii** and **iv**) close-ups of vFW and vHW. See Extended Data ( Sourakov, 2020) for details.

For *Vanessa atalanta* ( Figure 3), I only had access to two larvae which I collected as penultimate instars on nettles. Both resulted in spectacularly different wing patterns after being injected in the early pupal stage with ca. 0.5 ul of 2% heparin deep into the abdomen. For comparison, I provide a typical specimen from the collection (there’s little natural variation in the wild types in this widespread Holarctic species). This species is an excellent illustration of the established fact that wing pattern elements affected by heparin do not correspond to any particular color or wing position (see introduction in Sourakov & Shirai (2020) for review). In *V. atalanta*, it is the red marginal band that is expanded and obscuring the greatly reduced dHW eyespots. On forewings of this species, it is the white, red and blue colors that are expanded at a cost to the areas normally occupied by dark scales.

**Figure 4.**
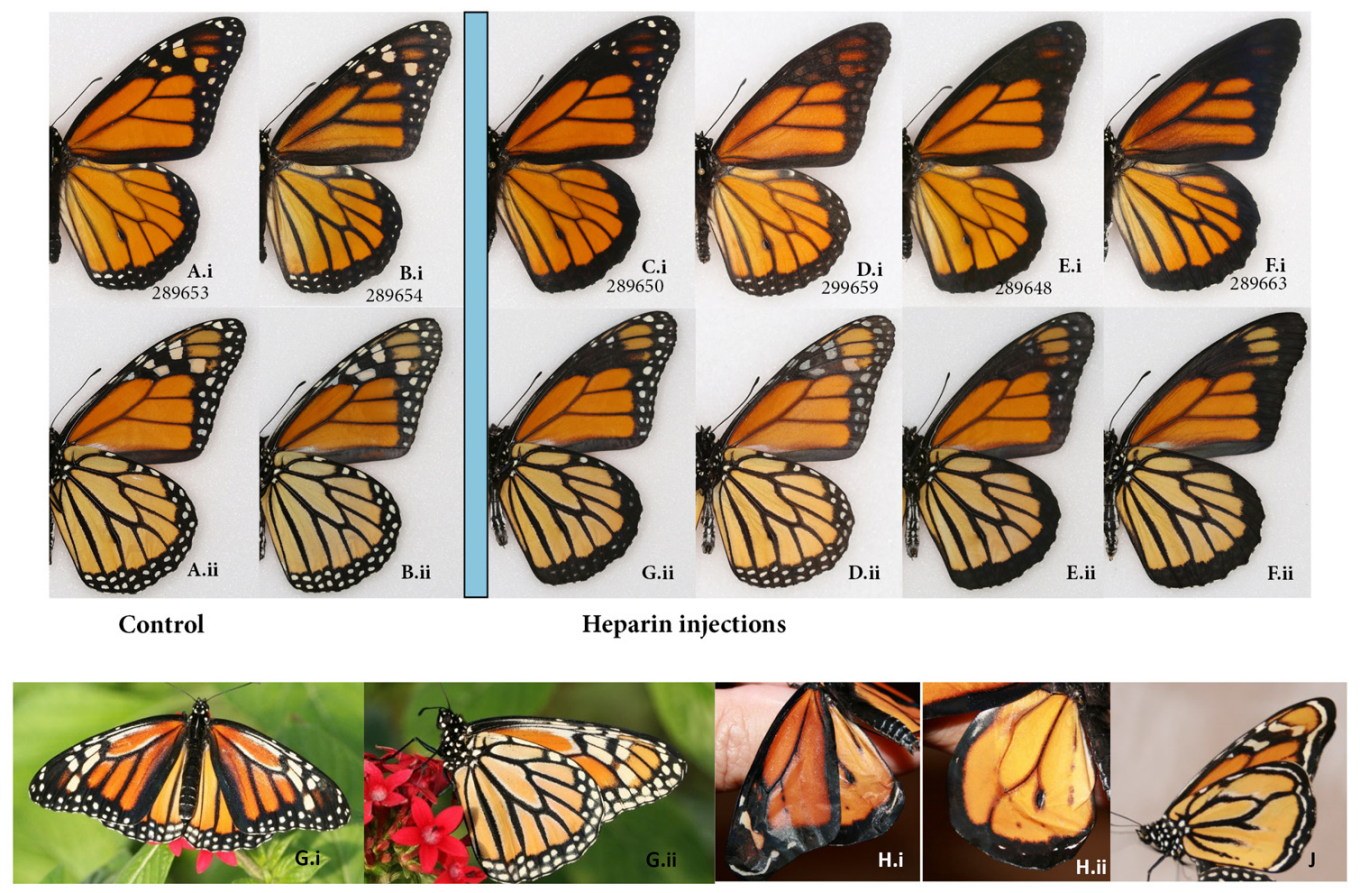
The monarch, *Danaus plexippus*. ( **A**– **B**) Wild type vs. ( **C**– **F**) heparin-induced wing pattern when injected as pupae or prepupae; dorsal ( **i**) and ventral ( **ii**) surfaces. ( **A**, **C**, **D**, **E**) males ( **B**, **F**) females. Aberrant monarchs, with extra white spots ( **G**) and without veins ( **H**, **J**), courtesy of Edith Smith. See Extended Data ( Sourakov, 2020) for details.

**DANAINAE:** ***The monarch, Danaus plexippus.*** The effect of maximum heparin-induced transformations of the monarch’s dorsal wing pattern is immediately obvious ( Figure 4). The checkered pattern, so characteristic of wing margins in the wild type, becomes uniformly black. Neither the black pattern corresponding to the venation, nor the ground color is affected in the process, and the border does not expand past its normal width.

Two trials with this species were conducted in October-November 2019 and March-April 2020. In the first study involving 42 individuals, 32 prepupae and pupae were injected with varying concentrations and quantities of heparin. The second trial on 57 individuals, 44 were injected as pupae with the aim of narrowing the sensitive time window and dosage within that stage.

If one is to consider less transformed individuals (e.g., Figure 4C, E, F) in which the change is intermediate between wild type and the extreme levels of transformation, one would realize that the loss of checkered pattern of the wing margins originates with the dHW, then progresses into the dFW and then to the vFW, and that proximal FW spots are eliminated before the distal ones. Increasing the heparin dosage past a certain level, while affecting the ability of butterflies to eclose and spread their wings, did not alter the pattern any further. However, there is an exception to the general pattern of transformation when it comes to the individual injected later in the pupal stage ( Figure 4D). This individual shows a practically normal underside, but the upperside margins tainted by a veil of dark coloration, with the spot pattern barely visible. For a complete set of photographs and associated injection data one is referred to the Extended Data ( Sourakov, 2020).

**Figure 5.**
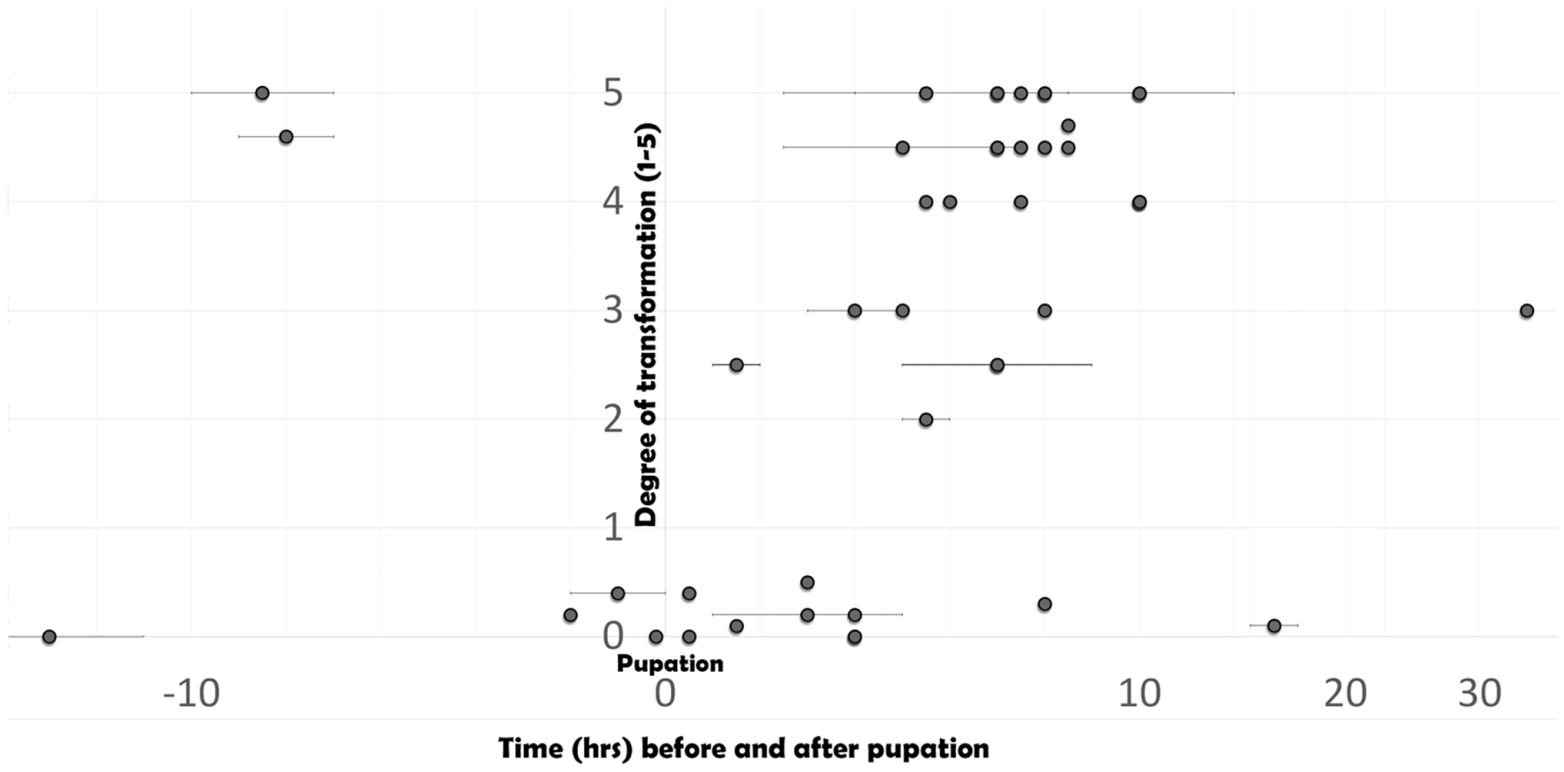
The monarch, *Danaus plexippus*. Degree of transformation (as determined by a blind score assignment) in relation to the time before or after pupation. See Extended Data ( Sourakov, 2020) for injection details.

By blindly assigning scores from zero to five (with five being highest degree of transformation, and zero corresponding to controls), I evaluated the timing during which the heparin action is the strongest in relation to the moments of pupation ( Figure 5). It is clear that during the time immediately surrounding pupation, the transformation is difficult to achieve, while for injections made 7–10 hBP or 7–10 hAP, the transformation peaks. There is limited data on the window between 10 and 40 hAP – the individual that was transformed (though differently) at 32 hAP ( Figure 3D) came as a surprise, as previous experiments with other species did not produce transformations after 24 hAP (e.g., Serfas & Carroll, 2005; Sourakov & Shirai, 2020, the present study).

The monarch is one of the best-studied species of animals in the world, from being the first butterfly to have its complete genome sequenced and annotated ( Zhan *et al.*, 2011) to its migration “demystified” by multi-faceted approach ( Reppert & de Roode, 2018 and references therewith). Current work on monarchs still involves analysis of big data from tagging information ( Taylor Jr. *et al.*, 2019) but is now supplemented with sophisticated techniques from neuroscience and genomics (e.g., Heinze *et al.*, 2013; Lugena *et al.*, 2019). The modern techniques of butterfly wing pattern research, such as CRISPR, also have been applied to the monarchs ( Mazo-Vargas *et al.*, 2017). In the latter article, in addition to muting *WntA*, ** the team from Harvard demonstrated that throughout the wing there was a loss of interveinous patterns in the monarchs. Authors characterize monarchs “as an example of extreme divergence from the nymphalid ground plan” (p. 10705) and conclude that *WntA* was “repurposed for the patterning of vein-contouring markings in monarchs” (p. 10706). In a parallel test, the authors demonstrated a loss-of function response (ectopic white scales) from an injection of 40 ug of dextran sulfate ( Mazo-Vargas *et al.*, 2017, p. 24 of suppl. Mat.). Surprisingly, the gain-of-function experiments (injection of heparin) appear to have not been conducted on monarchs prior to the present study.

Results presented here suggest that the MBS is affected by heparin. As no other effects were observed, it is hypothesized that all other systems of the nymphalid ground plan disappeared in monarchs over the course of evolution. This is in sharp contrast with what is observed in more derived nymphalids, such as the emperor and the gulf fritillary (see below). The abovementioned CRISPR experiments (backed up by *WntA* expression) seem to support this, as the muting of *WntA* and the corresponding loss-of-function pharmacological test had only the modest effect of white scales appearing at the vein junction surrounding the HW discal cell, perhaps reflecting the minor role of *WntA* in wing pattern development for this species.

Stability of the monarch’s general pattern may be of a strong adaptive significance, as this species relies on its aposematic coloration for signaling toxicity to predators. While many researchers discussing the nymphalid ground plan mention the role of veins in determining the position of the interveinous pattern elements (e.g. eyespots), the scales immediately adjacent to veins that produce such distinctive pattern as that of the monarchs and viceroys seem to have never been discussed. The present study, as well as the abovementioned CRISPR experiments, highlight the stability of this pattern element in the monarch. While we do not know the developmental mechanisms underlying this pattern formation, one can guess at the adaptive significance of such stability: since the spectrum of palatability is wide in this aposematic species, with some of the individuals being mimics of the others ( Alonso-Mejia & Brower, 1994), deviation from the established pattern would be detrimental to the population as a whole. Additionally, the migratory strategy employed by many populations of monarchs may have a stabilizing effect on the wing pattern: wing-related thermoregulation (e .g., Tsai *et al.*, 2020) and communal behavior at overwintering colonies have undoubtedly contributed to shaping the wing pattern.

In the present study, both prepupae and pupae of monarchs were tested with heparin injections with similar results, but minor differences should be noted. For one, the prepupae required more heparin (6% and 12 %, 1 ul) to achieve similar level of transformation as were obtained by injecting pupae, for which 3% concentration of the same volume was required. This can be partially explained by the change in weight that monarchs and other Lepidoptera undergo during metamorphosis (see below). Also, while wing pattern in the individuals injected as pupae was always symmetrical, one of the individuals injected as prepupae resulted in an asymmetrical individual (#289664, Extended Data ( Sourakov, 2020)), with one side transformed to a greater degree than the other. These occasional asymmetrical transformations seem to occur only when the prepupal stage is injected – see other examples: *Automeris io* (Figure 2 in Sourakov, 2017), *Heraclides cresphonetes* (Figure 5 in Sourakov, 2018b). These cases are puzzling, as wing patterns are under central control and changes should happen in a bilaterally symmetrical manner. One possible explanation is that at this stage, when wing veins are perhaps not yet fully formed, they are asymmetrically affected by the injection. Since veins play a role in wing pattern mapping, their asymmetrical development in turn leads to asymmetrical wing pattern formation. The degree to which the wing pattern formation in the monarchs is affected by the veins is demonstrated by Figures 4H–J, that shows vein-less individuals that emerged in a captive colony of the Shady Oaks Butterfly farm in Florida. In the same place, an aberrant individual of monarch with extra white spots emerged probably demonstrating the loss-of-function effect within the Wnt ligand ( Figure 4G).

**Figure 6.**
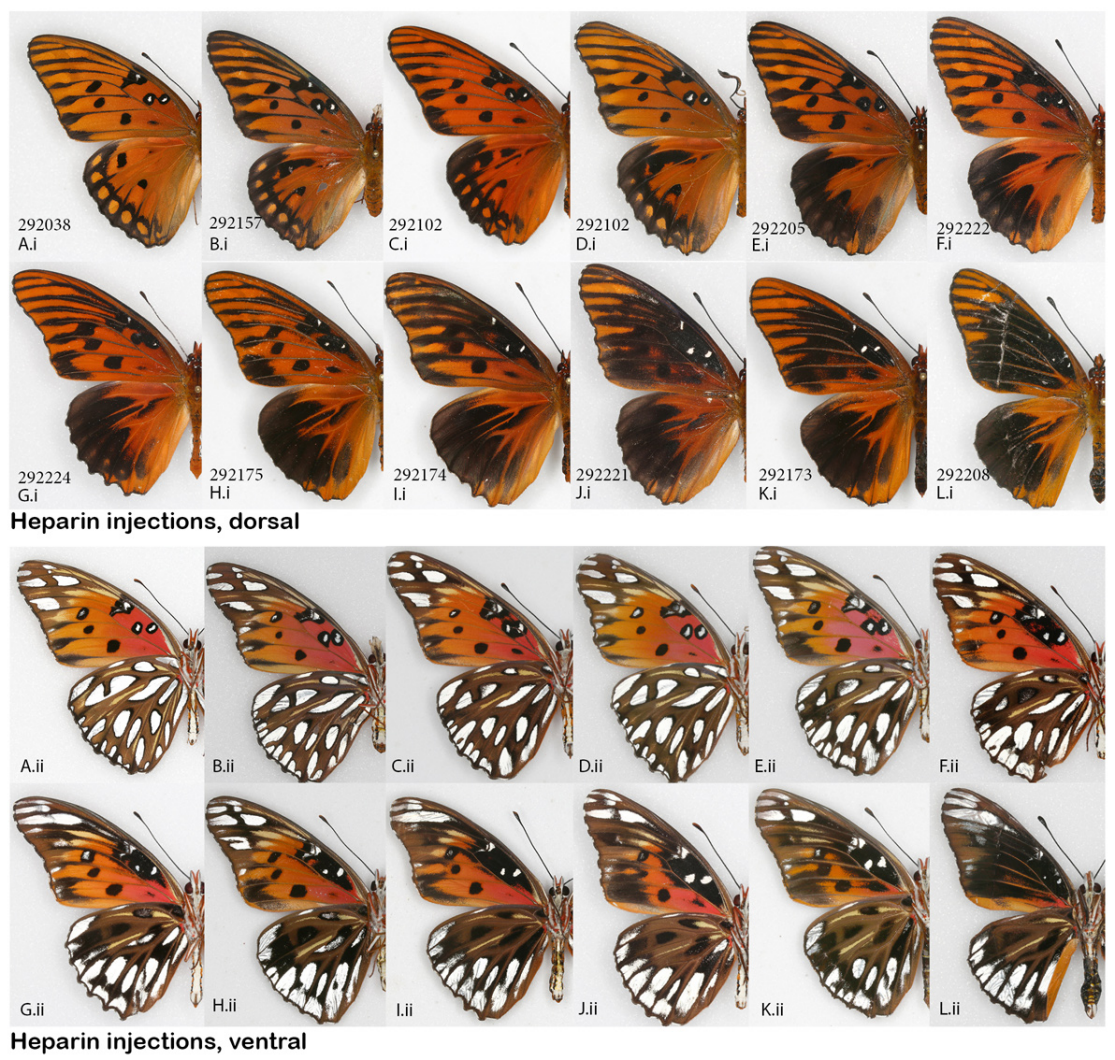
Heparin-induced wing pattern changes in the gulf fritillary, *Agraulis vanillae* when injected as prepupae ( **A**, **B**, **D**, **E**, **J**– **L**) or pupae ( **C**, **F**– **I**); ( **i**) dorsal, ( **ii**) ventral surfaces. See Extended Data ( Sourakov, 2020) for details.

**HELICONIINAE:** ***The gulf fritillary, Agraulis vanillae and the zebra longwing, Heliconius charithonia.*** In Figure 6, twelve specimens obtained as a result of heparin injections into prepupae and pupae are illustrated in order of increasing magnitude of the induced transformation. The changes consist of expansion of melanic territories dorsally, and some of the silver spots ventrally, with a number of silver spots, however, disappearing basally. As with the monarchs, both prepupae and pupae were tested with somewhat similar results. Among 10 strongly transformed individuals as the ones shown in Figure 6F–L, four were injected during the period 4–6 hAP, while the other six were injected 0–24 hBP as prepupae. Heparin solution of 3–4% at volumes of 1–2.5 ul (0.03-0.1 mg) are sufficient to induce strong transformations in the wing pattern without being lethal (see Table S1 ( Sourakov, 2018c) and Extended Data ( Sourakov, 2020).

As with the monarchs, the gulf fritillary has been one of several model organisms in recent investigations into wing pattern formation ( Martin & Reed, 2014; Mazo-Vargas *et al*., 2017). Martin & Reed (2014, p. 376) showed how heparin injections result in the expansion of *Wnt*-positive patterns; transformations resulting from their 10-ug injection correspond to transformations in the present study, as shown in Figure 6E. Specimens in their study resulting from 20-ug heparin injection correspond to the present study’s specimens shown in Figures 6G–I for the dorsal surface and to Figure 6J for the ventral (although expansion of silver markings on the FWv is much less dramatic). It is interesting to compare these images to the results obtained using CRISPR to mute *WntA* ( Mazo-Vargas *et al*., 2017, p 10704). While in the present study we observe gradual expansion of *Wnt*-positive elements and reduction or loss of *Wnt*-negative patterns, in the *WntA* mKO specimens, the opposite effect expectedly happens – the elements that are lost here (e.g., basal silver markings on FWv) are greatly expanded and merged, and the elements that are gained from heparin injection (e.g., FWv border silver markings) are completely lost. At the same time, the unpublished cis-regulatory knockout of other genes by the same lead author resulted in specimens that are identical to the most transformed ones by heparin in the present study (see the twitter communication by Reed (2020)).

According to Martin & Reed (2014, p. 375), the heparin injections may affect not only *WntA* expression, but several other signaling molecules. Thus *A. vanillae* provides an interesting model species for further research, as there are numerous pattern elements that are affected, and expression and CRISPR data already exists for *WntA*.

It is fascinating that pattern elements that are as unique and uniform as the ventral silver spots in this species have diverse origin and are under different developmental controls. They result, in the words of Martin & Reed (2014), from “dislocated elements that diverged from the groundplan organization.” It is very likely, based on this and other studies, such as the recent comparative study of saturniid eyespots ( Sourakov & Shirai, 2020) or mimicry rings in *Heliconius* ( Concha *et al.*, 2019), that this scenario of convergent evolution of wing pattern elements is more common than we suppose. Tools currently available to us (including heparin injections) can shed light on the evolution of this convergence. Our results with papilionids (see below) also support this idea.

*Agraulis vanillae* provided a convenient subject for observing a gradual heparin dose-dependent change in the degree of pattern transformation. This enabled us to test which developmental stages are most sensitive to treatment and lead to greater modification of wing patterns and whether temperature has an effect on the degree of transformation. Eleven successful manipulations, arranged in order of magnitude of effect on wing pattern, can be correlated with injection stage and dose by consulting Extended Data ( Sourakov, 2020). This wing transformation spectrum is contrasted with normal wing patterns that were not altered by injection at 1HBP ( Figure 6A).

Martin & Reed (2014) injected their *A. vanillae* at 10–16 hAP, so the more modest expansion of silver markings on the vFW in their case may be explained by the difference in the timing of the injections: in the present study, similar levels of transformation resulted when injections were done either before pupation or 3–6 hAP. This corresponds to observations on common buckeyes (see above), where prepupal injections seemed to have a similar, but less pronounced effect on some pattern elements than the pupal injections, perhaps due to differences in the timing of their development.

It should be noted that unlike a wide variety of buckeye phenotypes in conspecific and congeneric populations, among which one can find many parallels to the effects of heparin, the pattern of gulf fritillary seems to be naturally quite stable within a species. After searching the extensive MGCL collection, only one wild-collected aberrant individual similar to Figure 6I, and another less transformed (similar to Figure 6D) were found among over 1000 pinned specimens ( Sourakov 2018c, Figure S2). Hence, such aberrations are very rare in nature: I am aware of just three additional individuals (see thread in Reed, 2020). While there is an opinion that heparin mimics the cold-shock action, and that these wild aberrant individuals were possibly cold-shocked as pupae, I think mutation is the more likely cause: if the cold-shock were responsible, we would have seen such aberrations much more frequently in this very common and widespread species. Whatever the reason, the very existence of wild-caught specimens with this phenotype is of significance for understanding selection mechanisms that lead to divergence of wing patterns in Heliconiinae. They suggest that a natural mutation (and not only artificial pharmacological or CRISPR disruptions) can cause them, and thus that these changes represent real material for evolution.

In the closest relatives of *Agraulis*, which some would consider congeneric, such as the Mexican silverspot, *Dione moneta*, the border silver markings of vHW and the basal black markings of vFW are expanded, invoking associations with the present results of heparin injections. Moving in the opposite direction, the julia butterfly, *Dryas iulia* (also very closely related to *Agraulis*), lost not only all its silver markings but also most of its melanic patterning, as if the *WntA* and perhaps several other genes have been “muted” by evolution, leading to a simpler pattern of red with a narrow black border.

**Figure 7.**
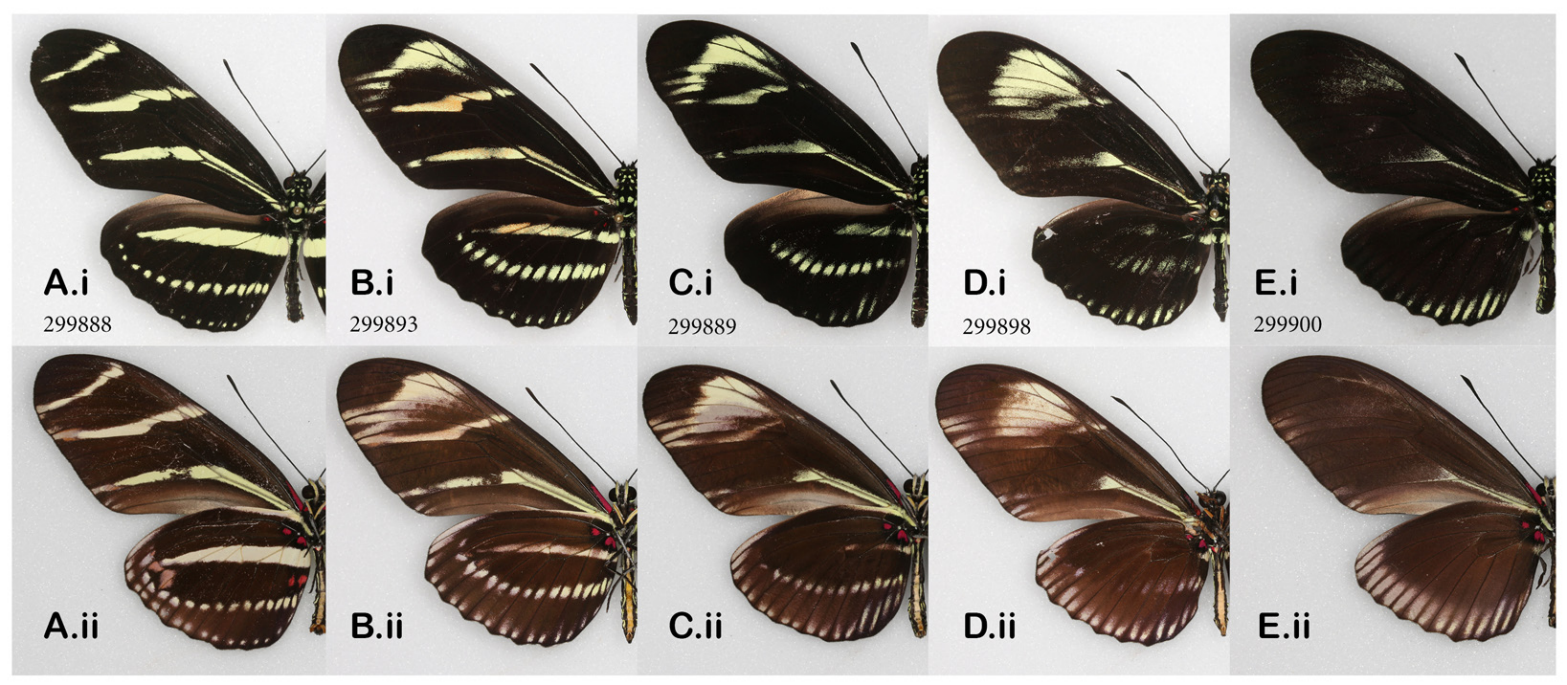
Heparin-induced wing pattern changes in the zebra longwing, *Heliconius charithonia* when injected as pupae; ( **i**) dorsal, ( **ii**) ventral surfaces. See Extended Data ( Sourakov, 2020) for details.

As for the zebra longwings ( Figure 7), the heparin action there presented few surprises after considering other species in this study: the MBS elements (black on HW, black and yellow on FW), expanded together with the discal spot elements, first pushing proximally then obscuring yellow pattern elements, with the subsequent complete overriding of the pattern as in Figure 7E. In many ways, the effect observed in this species is congruent with that of other nympahids (with the exception of the monarchs). All nymphalids demonstrate homologies between pattern elements such as border and discal elements, despite differences in their shape and color. At the same time, zebra longwing transformations underscore the striking contrast with papilionids: its black-and-yellow striped pattern and similarly highly patterned yellow-and-black aposematic coloration of the giant swallowtail react completely differently to heparin, showing that patterns of these two species has little in the way of underlying homologies. The best time to inject the pupal stage of the zebra longwing, based on 14 injections made here, is, like in the monarchs, seems to be between 7.5 and 11 hAP.

**Figure 8.**
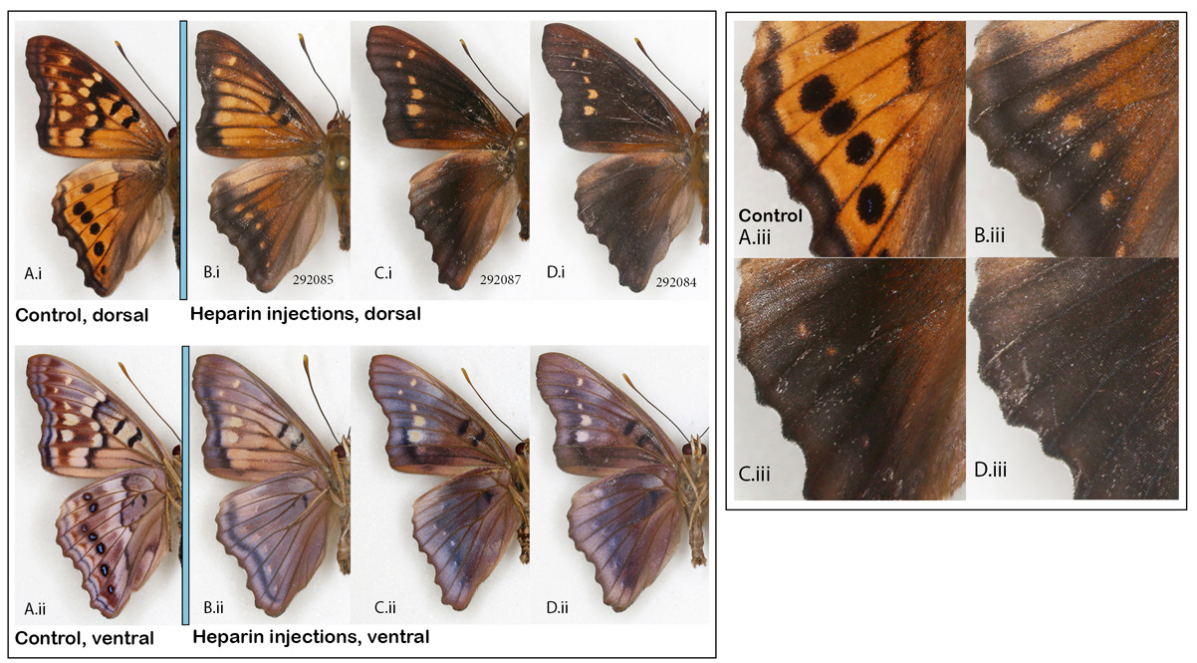
Normal wing pattern ( **A**) vs. heparin-induced wing patterns ( **B**– **D**) in the males of the tawny emperor, *Asterocampa clyton*; ( **i**) dorsal, ( **ii**) ventral surfaces, ( **iii**) close-up of HWd. See Extended Data ( Sourakov, 2020) for details.

***The tawny emperor, Asterocampa clyton.*** When I reported the successful transformation of a specimen of the tawny emperor butterfly by heparin injection ( Sourakov, 2018a), that single female individual, in which the dorsal surface of the wings turned almost entirely black, seemed to represent the furthest possible divergence from the normal phenotype. This result is replicated again here in a male specimen ( Figure 8D), with more accurately measured data concerning the injection. In addition to replicating the previous results, the main purpose of adding this species to the mix here was to obtain intermediates between the normal pattern and the most extreme transformations. Variation in resulting phenotypes was achieved by lowering the dose of heparin and using the prepupal stage in addition to the pupal stage. As a result, two intermediate transformations were achieved ( Figure 8B, C): the first injected as a prepupa ( Figure 8B) and the second as a pupa ( Figure 8C). The individual in Figure 8C is not drastically different from the one in Figure 8D, even though the former was injected with three times less heparin than the latter. The amount of heparin injected into the prepupa ( Figure 8B) was between the doses received by the other two transformed individuals.

It is interesting to note that the dorsal hindwing border spots, normally black in this species, rather than disappearing into the black background, as happened in maximally transformed specimens, appeared as orange spots in two of the intermediates ( Figure 8(iii)). Marginal eyespots in many Nymphalidae are concentrically organized and serve as models for studies of development, as for example, in *Bicyclus anynana*, where they have been shown to be positively regulated by *Wingless* ( *Wnt*) ( Özsu *et al*., 2017). *Wnt* signaling delimits the boundaries of wing spots, as reviewed by Martin & Courtier-Orgogozo (2017), and is affected by heparin. Figure 8(Aiii–Diii) suggests that, even though the serial border spots of the tawny emperor are not as concentrically organized as in many other nymphalids, they are nevertheless homologous. The marginal bands migrating basally under the influence of heparin are inhibited from invading eyespot centers. One can speculate that these eyespots, like the more concentrically organized ones, are also formed via a signal produced by the few eyespot-organizer cells in the eyespot’s center, as was first described by Nijhout (1980) and recently studied histologically by Iwasaki *et al*. (2017).

The eyespots of nymphalids have attracted much research, and the results obtained herewith on *Asterocampa* support some (e.g., Martin & Reed, 2014) and contradicts others (see review of the version 1 of this paper by Martin (2018)).

**PAPILIONIDAE**

***The giant swallowtail, Heraclides cresphontes, the spicebush swallowtail, Pterourus troilus, and the polydamas swallowtail, Battus polydamas.*** Two of the species tested here belong to the tribe Papilionini, but are from separate clades: *Heraclides*, which feeds as larvae on Rutaceae, and *Pterourus*, which favors Lauraceae) ( Aubert *et al.*, 1999). The third species is from the ancestral tribe Troidini, which has been separated from the Papilionini for a prolonged period of time, comparable to the evolution of many major Lepidoptera families ( Condamine *et al.*, 2018), and that includes such iconic species as birdwing butterflies.

Since the giant swallowtail is a contrastingly patterned species, one would expect substantial changes to its pattern resulting from heparin injections, if one is to base one’s expectations on prior experiments with nymphalids. Similarly, based on nymphalids, the highly patterned border of the spicebush swallowtail, consisting of turquoise spots, can be expected to be transformed (e.g., reduced in size and overrun by expanding marginal bands if they were remnants of eyespots, or, on the contrary, lose definition and get “smudged” towards the wing base, if they were part of MBS).

**Figure 9.**
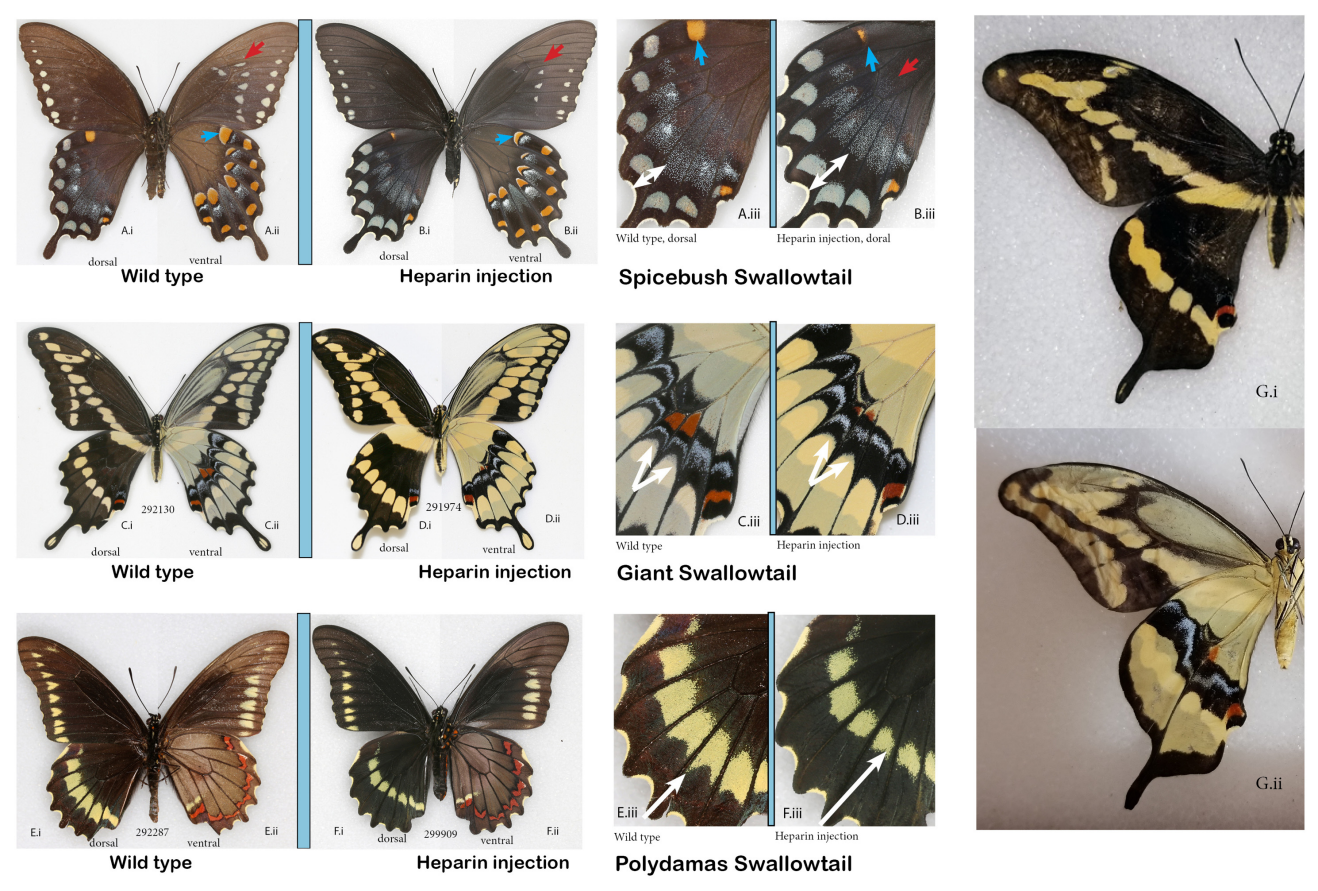
The normal ( **A**) spicebush swallowtail, *Pterourus troilus* ( **C**) giant swallowtail, *Heraclides cresphontes* and ( **E**) polydamas swallowtail, *Battus polydamas* ( **E**) vs. the corresponding individuals that underwent heparin injection as pupae ( **B**, **D**, and **F**). ( **G**) Aberrant giant swallowtail without veins, courtesy of Edith Smith. ( **i**) dorsal, ( **ii**) ventral surfaces and ( **iii**) close-up of hindwings focusing on the differences. See Extended Data ( Sourakov, 2020) for details.

These expectations, however, proved to be far from the case. To see the heparin-induced changes in these two species, one needs to zoom in and examine thoroughly the wing patterns of wild types while comparing them to the experimental specimens: in the spicebush swallowtail and in the polydamas swallowtail, the central elements of dHW pattern have shifted proximately under the influence of expanding black margins ( Figure 9A, C). Notably, the turquoise spots in the spicebush swallowtail remained unaffected, similarly to the dFW orange spots of the tawny emperor butterfly ( Figure 8CD), indicating a possible homology between these elements.

In the giant swallowtail, the heparin-induced changes manifested themselves only on vHW, where transformations were barely noticeable and were restricted to the highly-patterned band that runs across the center of the wing.

Koch & Nijhout (2002) determined that, in swallowtails, the wing margins serve as organizers of the wing pattern and that the veins serve simply as boundaries. This conclusion was based on the observed wing pattern transformation in an aberrant (vein-less) *Papilio xuthus* specimen, which also allowed the authors to hypothesize homologies among wing pattern elements.

It becomes quite clear, by comparing their study specimens and ours, that the vHW band of the giant swallowtail where the heparin-induced changes occurred is homologous to the wing margin elements of the nymphalid groundplan, and hence the observations made here are completely logical. In *P. xuthus*, the same pattern elements (including the red spots) are located much more marginally, but otherwise are very similar to these of the giant swallowtail.

These MBS elements are shaped and given their slightly unique positions by the veins; in the vein-less individual of Koch & Nijhout (2002), all these elements formed complete, uninterrupted bands akin to the band found in nymphalids. Here, for comparison, I included another illustration of vein-less specimen, this time of the giant swallowtail, reared in a captive colony of the Shady Oak Butterfly Farm ( Figure 9G) that shows uninterrupted bands both dorsally and ventrally, developed without being shaped by wing venation. In the giant swallowtail, based on the twenty surviving individuals that were injected with a variety of doses at different stages, it seems that one should also target the window around 5 hAP if one wanted to replicate these results.

The giant swallowtail is widespread and can be geographically variable in the characters that demonstrated heparin-induced changes ( Warren *et al*., 2013). The present study suggests that both the intraspecific variation in ventral hindwing bands and the variation among species found in the closely-related South American taxa, such as *Heraclides paeon*, *H. homothoas*, *H. melonius* and *H. thoas* ( Tyler *et al*., 1994 plate 89) would map onto the *Wnt* gene ligand. It also illustrates how wing pattern formation can be compartmentalized in some species, so that the variation in gene expression of pattern-mapping genes can affect only some of the compartments, but not others, and it may help to explain the existence of so many species sharing almost identical patterns in the New World.

A total of six individuals of the spicebush swallowtail were injected with heparin, and only two survived (both injected as pupae). There was a visible transformation of the wing pattern in one of them (injected 5HAP) ( Figure 9B). None of the numerous *P. troilus* specimens that I examined in the MGCL collection sported this phenotype, giving me confidence, despite limited sample size, that it was heparin-induced. The rare aberrations *flava* and *addenda* figured in Warren *et al*. (2013) might be explained by the results presented here.

In the polydamas swallowtail, injections were made to 22 individuals (20 pupae and two prepupae), among which all but two (one pupal and one prepupal injections) resulted in adult butterflies. The exact timing of pupation in relation to injection time was not obtained: pupation always occurred between 10 pm and 7 am, and the pupae were more than 3 hAP (based on degree of tanning) but less than 12 hAP. Of the surviving 20 individuals, 10 were injected with 4.5% heparin (volumes ranging from 0.5 ul (N=2) to 1.5ul (N=1). The other 10 were injected with either 9% heparin solution (N=8, volumes from 0.5 ul (N=2) or 1 ul (N=6)) or 18% heparin (N=2, volume 1 ul; the one pupa that did not survive the injection was of the latter kind). 

To evaluate pattern changes in the polydamas swallowtail, the Florida Museum collection was utilized, with 38 specimens collected in Florida randomly selected and photographed. Measurements were taken from these and the heparin-treated butterflies to calculate the ratio of the HW length (from base to the longest point along vein M _3_) to the width of the black border along the same vein as shown with white arrows ( Figures 9 E.iii and F.iii). Based on visual observations as well as statistical analysis (Paired T-test), there is a strong statistical difference between the experimental group and the wild types from the collection. Six individuals in the experimental group fell completely outside of the range of the control group, and five of them were injected with a higher concentrations (9 or 18%) of heparin than the rest of the cohort, which were injected with 4.5% solution. The latter fact unequivocally demonstrated that the observed difference was caused by heparin injections; it also demonstrated that the concentration that needs to be used (other things being equal) is much higher than in similarly sized monarchs, where 3% concentration was usually sufficient and above 6% was likely to be lethal. This interspecific difference between reaction threshold and tolerance to heparin runs across all of my heparin-related experiments, as well as those of others (e.g., Fenner *et al.*, 2020). The heparin-induced phenotype is nearly identical to that of *B. polydamas christopheranus* found in the Caribbean ( Warren *et al.*, 2013).

The evolutionary separation point between the two lineages examined here (Troidini and Papilionini) dates back around 40–60 million years ago (e. g., Condamine *et al.*, 2018), so the observed differences between them are not surprising. On the contrary, considering how long the two lineages have been diverging, there are seemingly more similarities than differences in the wing pattern organization, as can be determined by this very limited and crude experiment. The transformations that happen to the dHW of ** the giant swallowtail suggest that the MBS can “migrate” inwards of the wing all the way to the M2-M3 cross-vein. This, in turn, points towards the possibility that dHW eyespots of saturniid moths (such as *Automeris io* and *Antheraea polyphemus*), which are so strongly affected by heparin ( Sourakov & Shirai, 2020), may in fact, at least in part, be derivatives of the MBS.

**CRAMBIDAE**

**Figure 10.**
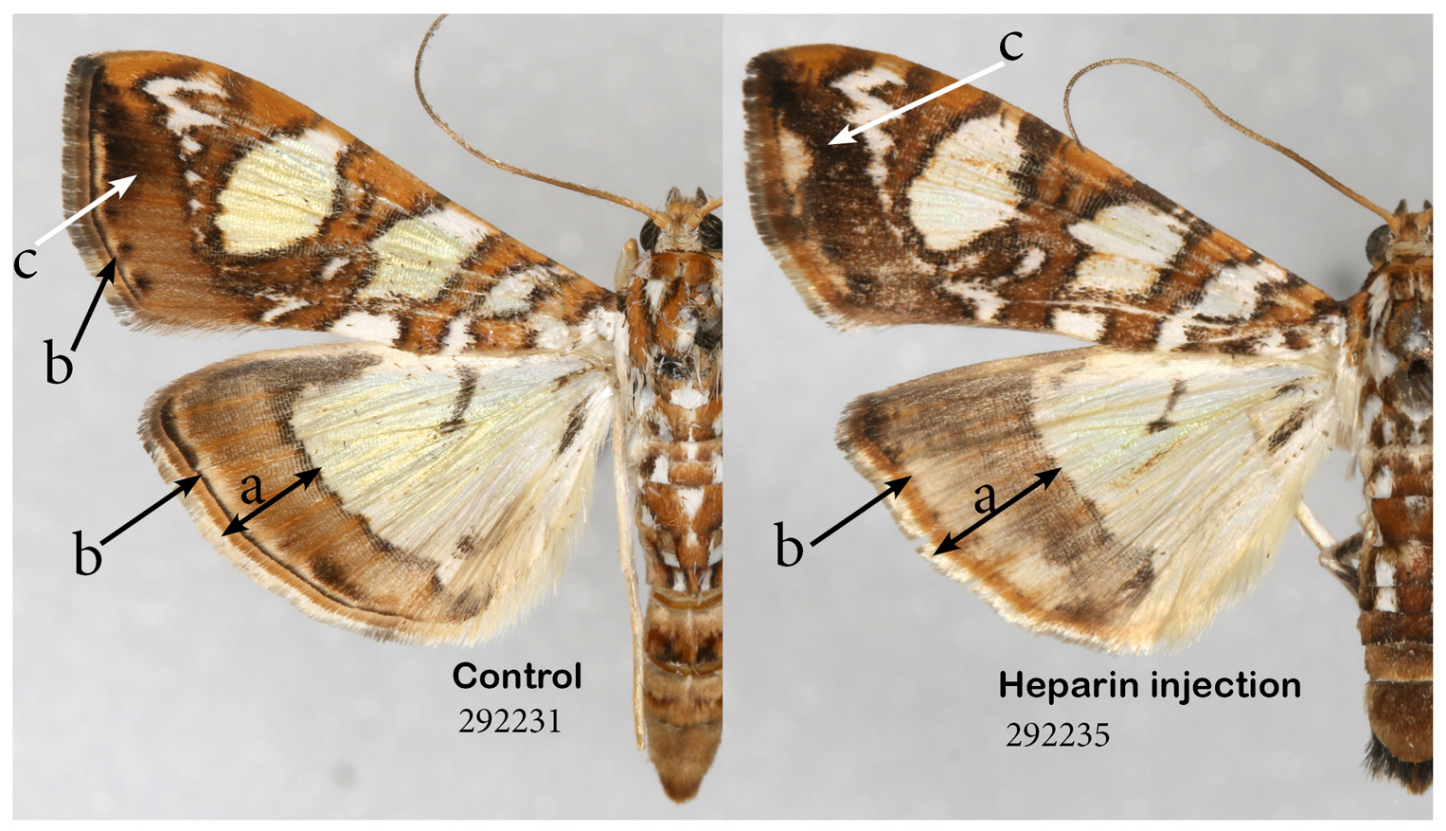
Normal individual of the mulberry leaftier, *Glyphodes sibillalis* (left) vs. heparin injected as pupa (right). Heparin causes expansion of the border area ( **a**). Heparin also causes expansion of marginal band and loss of definition in submarginal band ( **b**) and expansion of some of the melanic territories ( **c**). See Extended Data ( Sourakov, 2020) for details.

***The mulberry leaftier moth, Glyphodes sibillalis.*** This tiny species is relatively large by the standards of micromoths, but it still has a much smaller caterpillar, and hence my expectations of success were low. Nevertheless, among the two surviving experimental individuals, one injected with heparin as a prepupa, the other as a pupa, the latter proved to be different not only from the former, but also from the five control specimens. The changes are consistent with heparin-induced changes in, for example, nymphalids: the olive-brown hindwing marginal band expanded under the influence of heparin, and the normally thin and compact submarginal and marginal bands are also expanded and lost definition on both forewings and hindwings ( Figure 10).

**EREBIDAE**

**Figure 11.**
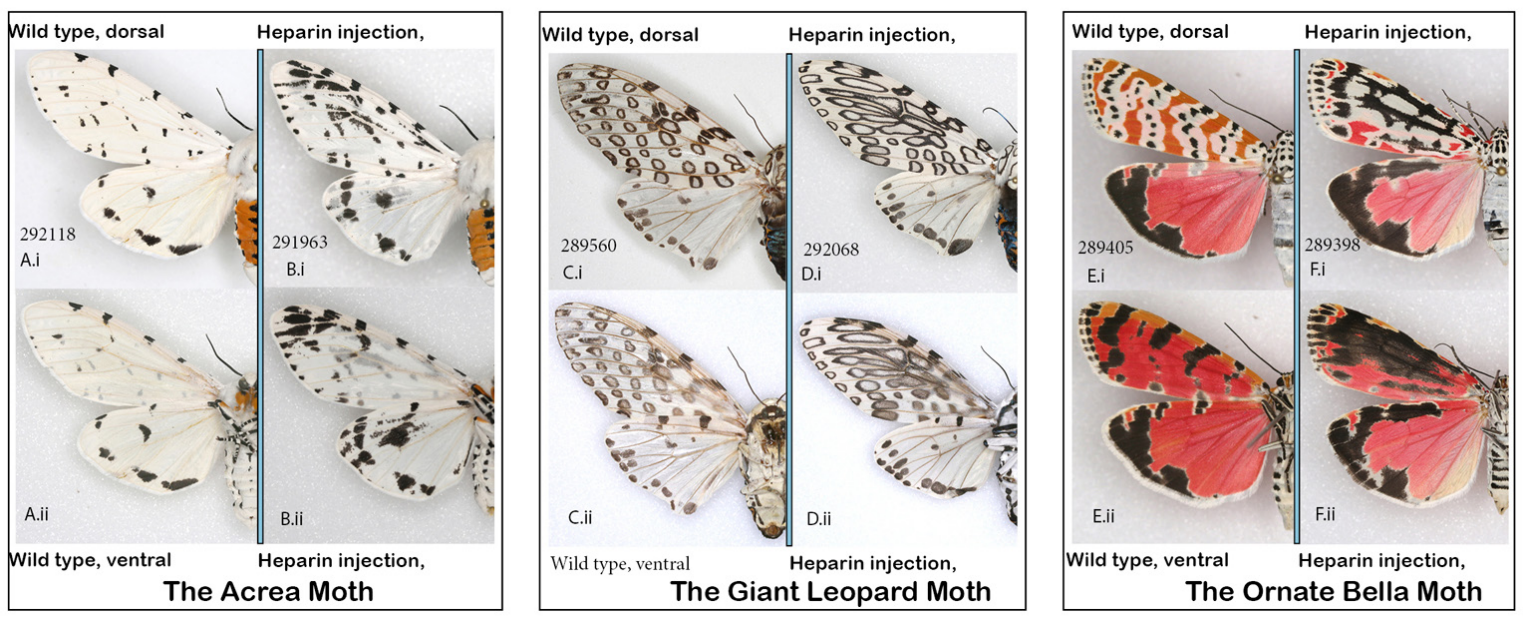
Heparin-induced wing pattern changes in three tiger moth species. ( **A**, **B**) The acrea moth *Estigmene acrea*: ( **A**) control; ( **B**) injected heparin as prepupa 11–14 hBP. ( **C**, **D**) The leopard moth, *Hypercompe scribonia*: ( **C**) typical wild-collected specimen; ( **D**) injected heparin as prepupa; ( **E**, **F**) The ornate bella moth *Utetheisa ornatrix*: ( **E**) control; ( **F**) injected heparin as pupa 12 hAP ( **i**) dorsal ( **ii**) ventral. See Extended Data ( Sourakov, 2020) for details.

***The leopard moth, Hypercompe scribonia, the acrea moth, Estigmene acrea, the ornate bella moth, Utetheisa ornatrix, and the fall webworm, Hyphantria cunea.*** While all four species examined here belong to the tribe Arctiini, *U. ornatrix* represents the Callimorphina clade, while the other three are relatively closely related within the Arctiina clade ( Zaspel *et al.*, 2014). There are clear differences between the two clades in wing pattern and in the ways in which they respond to heparin treatment ( Figure 11). Among the 40 individuals of the leopard and acrea moths that survived injection at different stages of development, obvious transformation was restricted to a single individual in each species injected as a prepupa within 12 hours before pupation (HBP), despite the fact that the doses and concentrations injected were quite high and timing varied widely. The heparin-induced changes were similar in their manner ( Figure 11). The alterations consisted of a very noticeable elongation of the black markings, to the point where some of the normally distinct spots sometimes merged, which happened both dorsally and ventrally. The expansion of individual spots was not consistent throughout the forewing, but was restricted to discal spot and the adjacent elements. A female of leopard moth injected 14 HBP showed the degree of expansion of hindwing marginal spots. I examined hundreds of individuals in MGCL collection but did not find any with similar phenotypes of acrea or leopard moth. For the fall webworm moth (not illustrated), I had only reared the white form without black spots. In the absence of any pattern, no heparin-induced changes were expected or achieved. The remote possibility that spots that are commonly found in this species would manifest themselves in heparin-injected all-white individuals did not come to fruition: 12 individuals injected as pupae were identical to the controls (N=40).

Overall organization of the arctiine wing pattern has recently been thoroughly treated by Gawne & Nijhout (2019) and Gawne & Nijhout (2020), who introduced “the arctiid archetype” (represented by a typical forewing pattern of *U. ornatrix*) and how the remarkable arrays of patterns found among tiger moths evolved from it. These authors divide the normal forewing of *U. ornatrix* into four elements. The two vertical bands of spots at the base of the wing (normally referred to as basal and antemedial lines) are the basal symmetry system (BSS). The discal spot (D) is always located on M2-M3 vein and the central symmetry system (CSS) consisting of two vertical bands of spots (the median and postmedial lines) are located on both sides of (D). Finally, the terminal band (T) and the van Bemmelen band (VB) are located between (CSS) and the wing margin. The latter elements are normally referred to as subterminal and terminal lines in taxonomic literature (e.g., Gordh & Headrick, 2001).

**Figure 12.**
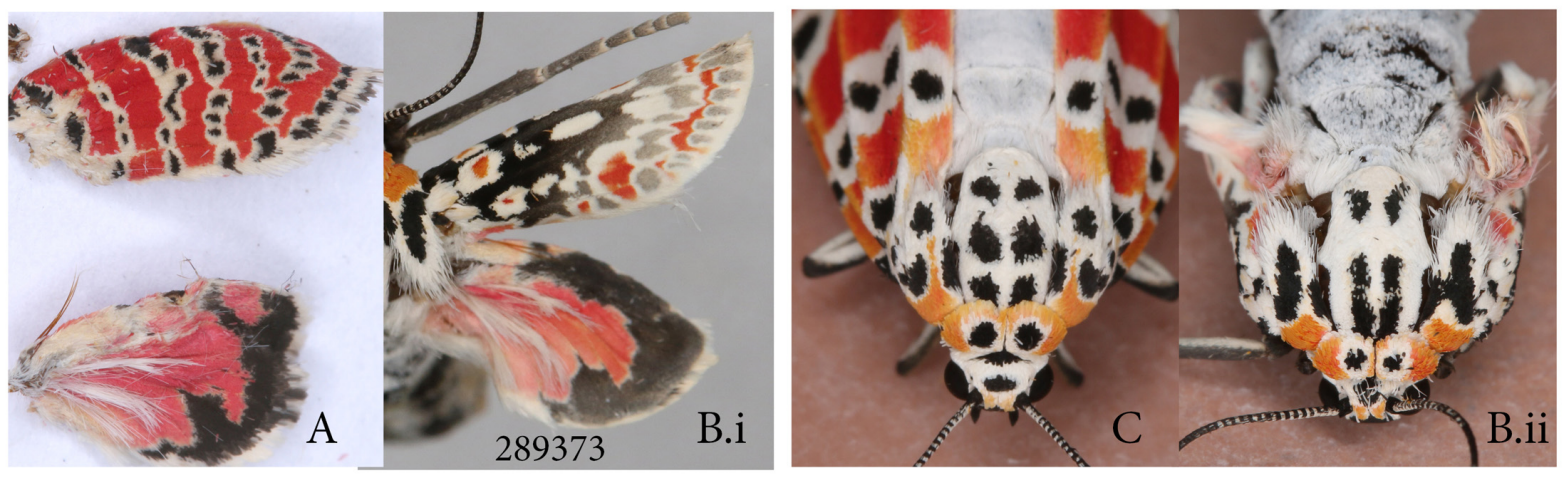
Maximum extent of heparin-induced wing pattern changes in the ornate bella moth, *Utetheisa ornatrix.* ( **A**) control specimen dissected out of a pupa, wings not spread. ( **B**) injected 0.5 ul of 3% (0.015 mg) heparin as pupa11hAP. ( **C**) typical specimen from the control group. ( **i**) dorsal ( **ii**) head and thorax.

**Figure 13.**
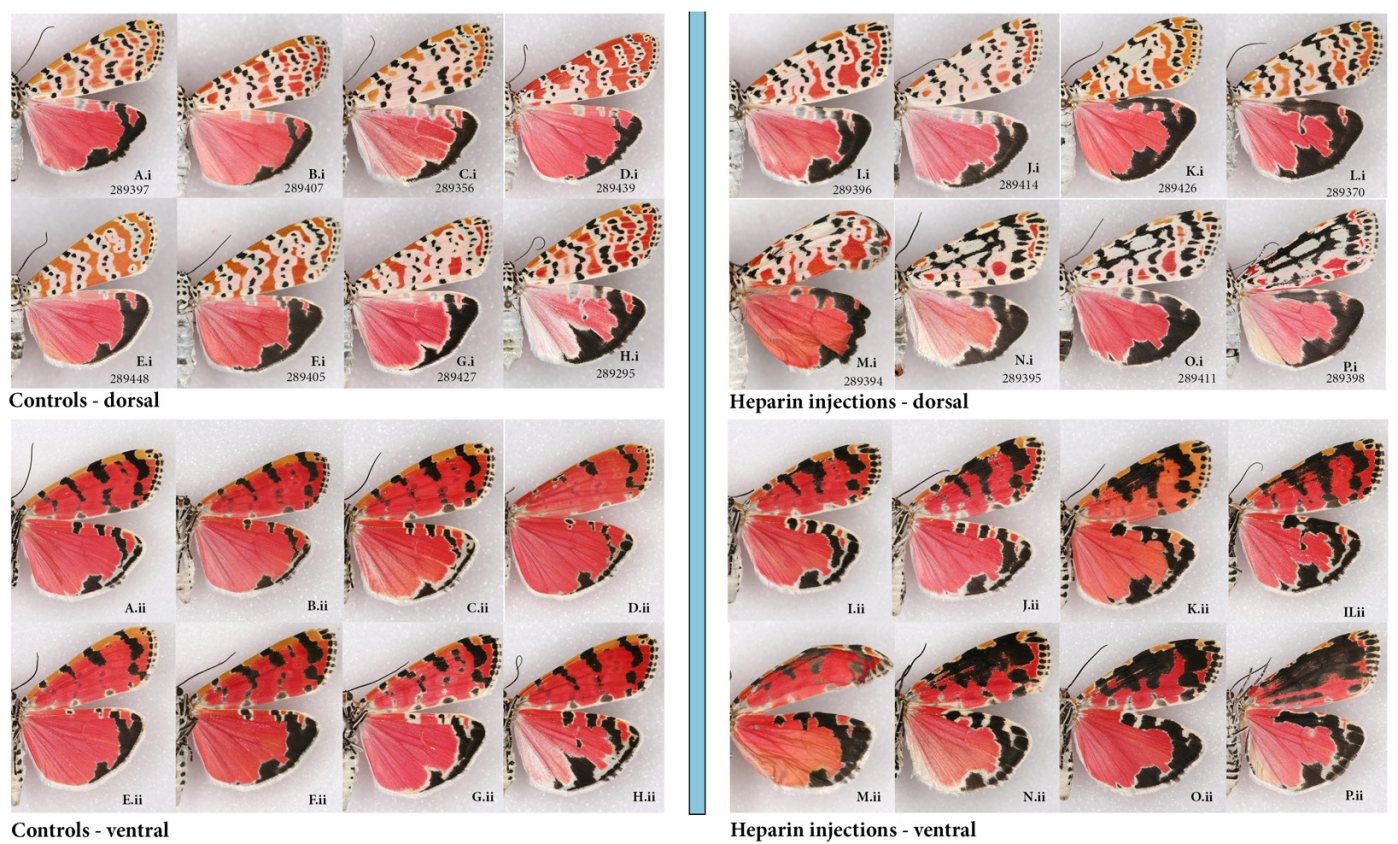
Heparin-induced wing pattern changes in the ornate bella moth, *Utetheisa ornatrix.* ( **A**– **H**) controls representing a variety of phenotypes from three broods ( **A**– **D**) males, ( **E**– **H**) females; ( **I**– **P**) injected heparin as prepupae ( **I** – at 6hBP, **K** – 9hBP, **M** – 11hBP, **N** – 8hBP) or pupae ( **J** – 14 hAP, **L** – 17 hAP, **O** – 19 hAP, **P** – 12 hAP). Figured in the order of increasing heparin effect. ( **i**) dorsal ( **ii**) ventral C ode following voucher # indicates brood # and sex (m-male, f-female). See Extended Data ( Sourakov, 2020) for details.

The ornate bella moth individual in Figure 12B demonstrates the maximum degree of transformation, in which FW pattern becomes almost a negative of its former self, with dark area occupying most of the wing. The ornate bella moth is the only species to date in which non-wing pattern elements can become affected: the black markings on the thorax and patangium ( Figure 12B.ii) have expanded, while the markings on the collar remained unchanged in this specimen and three other specimens shown in Figure 13 N–P. As with other species, at its maximum manifestation, the heparin-induced changes corresponded to the loss of viability, with the most extreme wing pattern transformations also correlating with the loss of ability to eclose and/or spread wings.

When continuous variation in heparin effects is considered in *U. ornatrix* ( Figure 13), it becomes clear that the effect is strongest around the discal spot of the forewing, followed by VB and CSS. It is also obvious that the white vertical bands that correspond to bands of black spots expand first, before heparin begins to affect the spots. The pattern is organized in transverse stripes but the expansion of pattern elements is directed predominantly along the FW length, so that the black dots and white stripes merge throughout the wing as the effect of heparin increases ( Figure 13I–P).

The ornate bella moths populations and individual broods exhibits a high degree of variability in the dFW with respect to the amount of white vs. red surface area, and the extent of dHW margin, including some sexual differences ( Figure 13A–H). Thus, at lower levels of heparin-induced transformation, the transformed specimens can only be reliably identified by their less-variable dorsal surface, where the black markings are also beginning to gradually expand under heparin-influence. When these ventral surface changes become substantial, they begin to correspond to the distortion of the black spots on the dFW (e.g., Figure 13K).

Among the three most transformed individuals that were able to emerge and spread their wings, the dFW became almost completely black and white with only a few splotches of orange left ( Figure 13N–P). Despite over 50 individuals surviving injections, the number of successfully transformed individuals was relatively low (see Extended Data Sourakov, 2020); the timing of corresponding injections suggests that 6–11 hBP in the prepupal stage and 8–17 hAP in the pupal stage are the periods when the wing pattern of the bella moth is most sensitive to heparin.

**Does heparin simulate cold-shock effect on wing pattern?**

While the bella moth is better known as a model species for studying chemical ecology (e.g., Conner, 2008; Sourakov, 2015a and references therewith), it also attracted the attention of biologists as an ideal model for exploring variation for at least a century (e. g., Pease, 1968; Remington, 1958). As was demonstrated by Charles Remington, they can be selectively bred from a wild stock to produce uniform lineages that diverge remarkably in their wing pattern ( Pease, 1968), but they also show relatively stable pattern within a local population and especially within a single brood (pers. obs.). Now that the bella moths have attracted attention as a possible model for exploring wing pattern development as a token “arctiid archetype” species, it will be useful to assess its wing pattern and its phenotypic plasticity from every possible angle.

**Figure 14.**
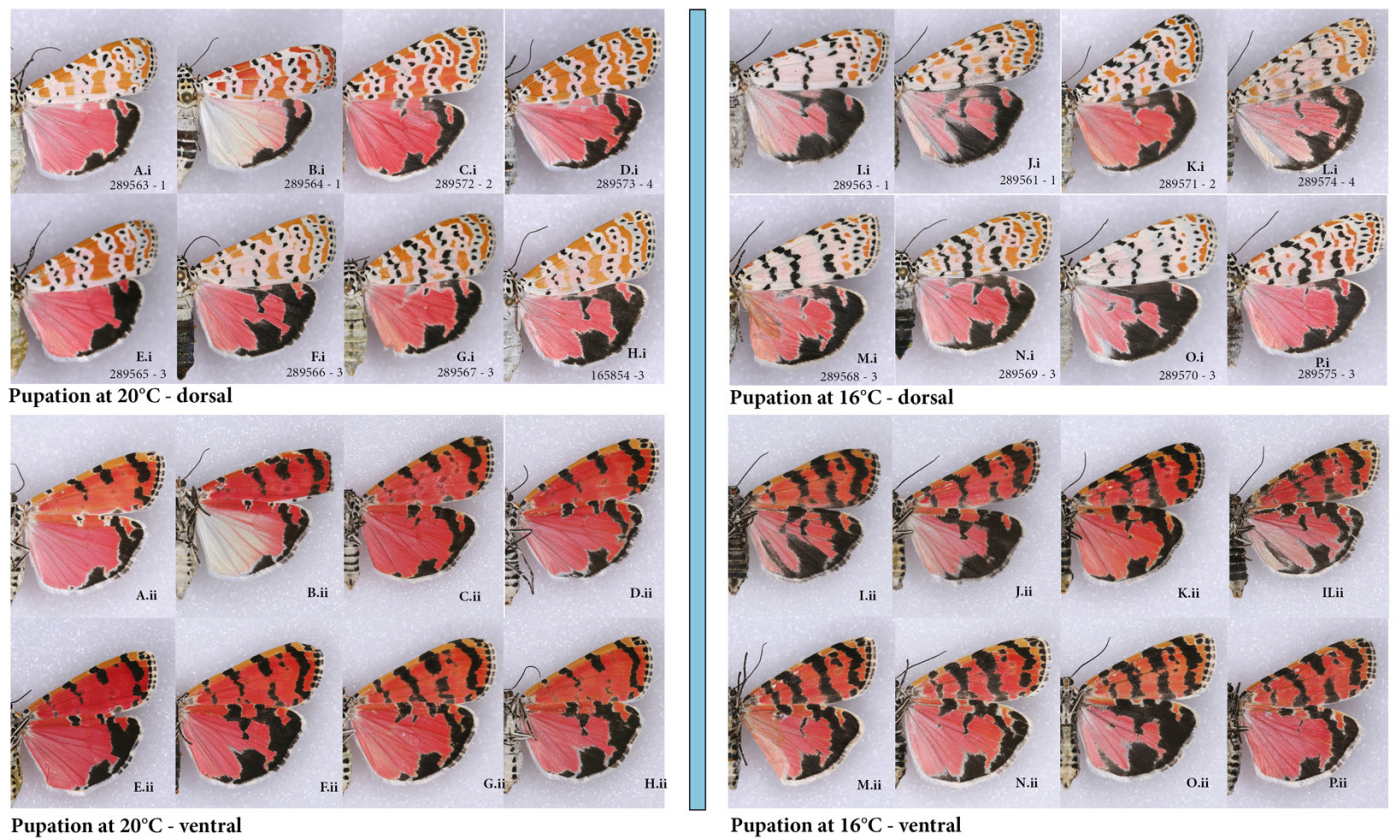
Cold-induced wing pattern changes in the ornate bella moth, *Utetheisa ornatrix*. ( **A**– **H**) raised at 20℃; ( **I**– **P**) raised in the last instar to adult at 16℃. ( **i**) dorsal ( **ii**) ventral; code following voucher # indicates brood #.

For instance, it has been suggested that heparin simulates cold shock effect, but is it equivalent to cold shock when it comes to specific changes it imparts on a given wing pattern? To answer this question, I illustrate specimens from several split-brood experiments, in which last instar larvae pupated either at room temperature of 22°C ( Figure 14A–H) or 16°C ( Figure 14I–P). This experiment is described in detail in Sourakov (2015b), and here only a few specimens, randomly chosen from several broods, are illustrated as an example.

While the results of cold-rearing experiment superficially resemble those of the heparin-injection experiment, the reduction of orange color yields its space to white background uniformly throughout dFW. Because of significant intrapopulational variability in dFW orange color, and because the expansion of black markings on dFW are minimal compared to all other wing surfaces, expanded bands on vFW are again the best indicators of whether cold temperature has an effect on the coloration of a particular specimen.

Cold-induced expansion of black markings happens uniformly in all directions on all wing surfaces. In contrast, heparin-induced expansions are non-uniform throughout the wing (strongest around discal spot, for example), happen along the wing’s length and are more pronounced on FW over HW. Presumably heparin acts by easing the flow of morphogens through the wing, and hence it is not surprising that there is directionality to the expansion of the affected wing pattern elements. Although there is a superficial resemblance between cold and heparin effects on phenotypes of the ornate bella moths, this resemblance is far from identical, and it is possible that the changes occur via different developmental mechanisms. Several other species, such as monarchs and gulf fritillaries, in which heparin causes expansion of melanic territories, showed no phenotypic changes when pupating in colder temperatures (pers. obs.).

## Appendix: Prepupa vs. pupa as a stage for manipulation of wing pattern

***Differences in survival by species.*** It is clear, from the species examined in the present study, that heparin treatments are tolerated differently by different species ( Table 1). This was also recently noted by Fenner *et al.* (2020). In the present study, very few experimental individuals among the tiger moths or monarchs died, and the polydamas swallowtail shows a degree of heparin tolerance remarkable for a butterfly of its size. In contrast, the zebra swallowtail had a zero survival rate, despite attempts to use low concentrations and dosage of heparin at different stages of development and at different conditions. In the giant swallowtail, despite injecting 60 individuals at different stages, visible transformation of wing pattern was difficult to achieve and only a third survived the injection. Within the Nymphalidae, survival sometimes varied between species and stages. The gulf fritillary and the monarchs were the most successful species, after the tiger moths, in their ability to survive heparin treatment, and both prepupae and pupae survived equally well. On the other hand, among the 100 buckeye individuals used in this experiment, the survival of heparin-injected individuals greatly depended on the developmental stage: 79% (n=19) of those injected as early prepupae and 45% (n=29) of those injected as late prepupae survived, but pupal injections did not yield any surviving adults. Despite lowering the dose and varying the stage of injection, as well as conducting injections at lower temperatures, achieving the survival of the tawny emperor was highly problematic: only 20% of the individuals survived injections, with 4 additional transformed individuals fully formed but unable to emerge from the pupa. It is possible that the species’ biology, for example their ability to sequester and/or detoxify certain secondary plant compounds correlates with their tolerance heparin injections.

***Is wing pattern transformation stage-sensitive?*** Heparin injections, for the most part do not interfere with pupation. Almost all of the individuals that were injected as prepupae and subsequently did not survive died in their pupal stage. It is certainly quite likely that, in most species, at least some wing pattern elements are laid down in the prepupal stage, and hence it is important to explore both prepupae and pupae.

The logical question becomes: does heparin linger from the time of injection onwards, or does its action correlate strictly with the time of injection? While the similarities in the outcome of late prepupal and pupal injections in the io moth ( Sourakov, 2017) may suggest that there is little difference whether a late prepupa or an early pupa is injected, it may not be the case in every Lepidoptera species. There seemed to be a different pattern of transformations in the buckeyes that were injected as pupae by Serfas & Carroll (2005) compared to the buckeyes injected as prepupae in the present study: here, the hindwing eyespots remained practically unchanged, throughout the transformation spectrum (except in one individual in which the pattern was completely overhauled, and which was not viable). Serfas & Carroll (2005), on the other hand, were able to gradually decrease the size of the dorsal hindwing eyespot by increasing the dose of heparin injected into pupae 5 hAP.

Among 36 heparin injections in leopard and acrea tiger moths, including many where the timing would have been perfect to achieve the transformation in Nymphalidae (ca. 5–8 hBP), the only obvious wing pattern transformations occurred in two individuals injected as prepupae ca.11–14 hBP. This suggests that, at least in these species, the effect is stage-sensitive. Another erebid, the ornate bella moth, in which 55 individuals were injected at a variety of developmental stages, demonstrated mixed results: among the four most dramatic transformations ( Figure 12, Figure 13), three were injected between 11 and 19 hBP, and one was injected 8 hBP. This corresponds to the results obtained in the monarchs, where the transformations were achieved more readily around 9–10 hAP and 8–10 hBP. Numerous observations from silk moths also suggest that injecting too early in the prepupal stage does not produce the visible transformations that can be achieved in late prepupal and early pupal stages ( Sourakov & Shirai, 2020).

***Heparin dosage, animal weight, and stages of metamorphosis.*** At the onset of these experiments, it was difficult to provide an accurate estimate of how much heparin was actually being delivered to the cells, because some heparin may have been lost as a result of ‘bleeding’ from the injection site. It has also become clear that the volume of heparin solution, in addition to its concentration may negatively affect the survival rates. Thus, for many species, I used smaller doses of more concentrated heparin injected deep into the pupal abdomen. With prepupae, the issue of loss of heparin remains, as deep injections cause acute reaction and frequent death, and thus injections need to be made subcutaneously.

**Figure 15.**
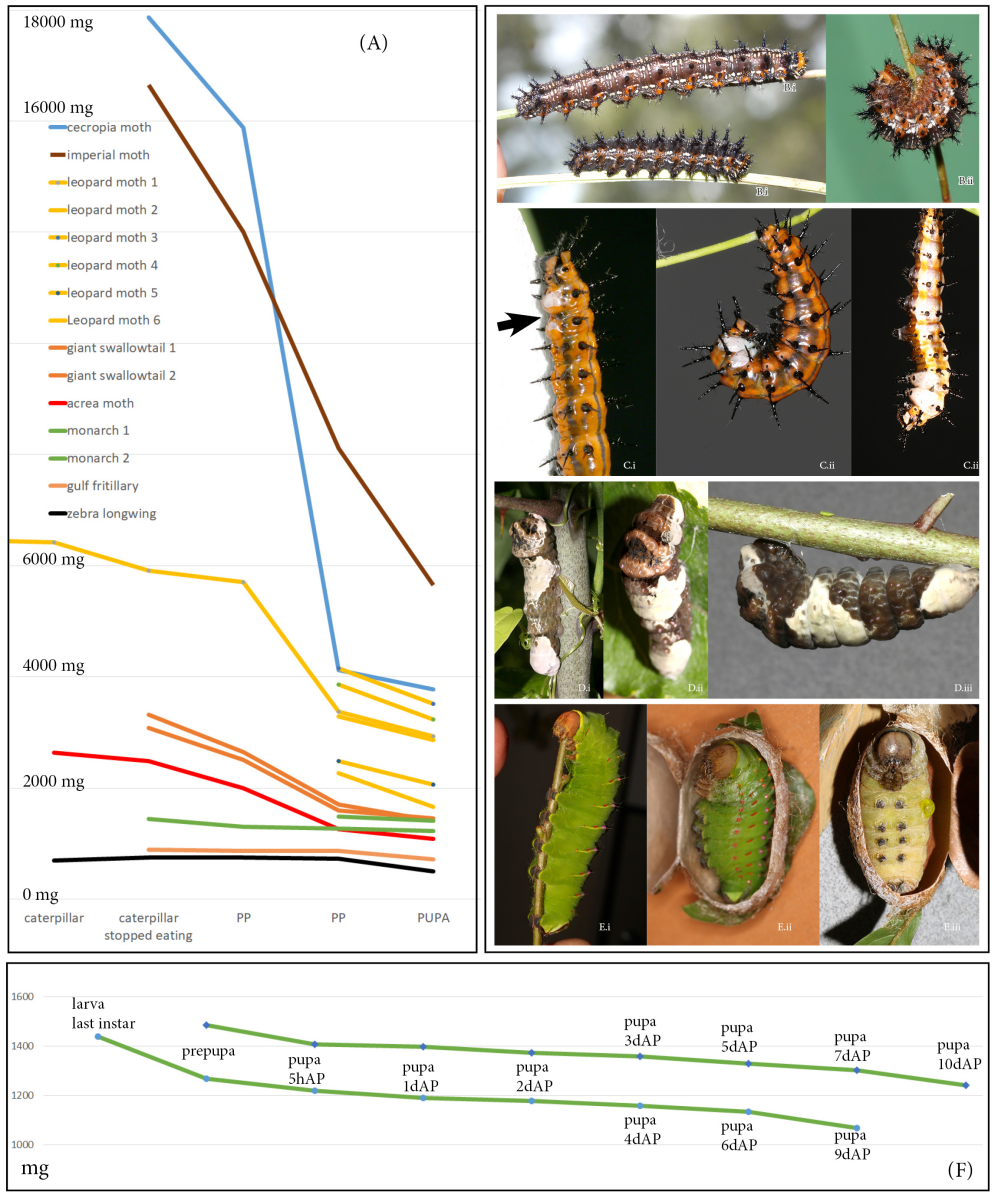
Weight loss by several representative butterfly and moth species during the transformation from late caterpillar stage to prepupa to pupa. ( **A**) Drop in weight from the moment caterpillars stopped eating until pupation in 8 different Lepidoptera species shows sharper decline in moths compared to butterflies. The slope corresponds to species’ biology: moths spinning more silk lose relatively more weight than their counterparts from the same family that spin less silk. Six leopard moth individuals are included to illustrate intraspecific variation. ( **B**) common buckeye, *Junonia coenia*; ( **C**) gulf fritillary, *Agraulis vanillae*; ( **D**) giant swallowtail, *Heraclides cresphontes*; ( **E**) polyphmemus moth, *Antheraea polyphemus*. ( **i**) caterpillar at the end of feeding, ( **ii**) early prepupae, ( **iii**) late prepupae. ( **F**) Decline of weight in two individuals of monarch, *Danaus plexippus*, from the end of feeding in caterpillar until day 10 of the pupal stage.

Another major consideration is the changes in weight that an animal undergoes during metamorphosis, as well as the intraspecific variation in size. My experience with the io moths, a species in which a normal female can be more than double the weight of a normal male, suggests that the transformation achieved by heparin injections is not only dose- and stage-dependent, but also depends on an animal’s weight/sex ( Sourakov & Shirai, 2020). I weighed immature stages of Lepidoptera species that I reared during the present study, plus two additional species of silk moths, the cecropia moth and the imperial moth, that I reared recently. Animals’ weights varied not only with species and within species, but also the same individual’s weight varied greatly depending on the stage of its development ( Figure 15). The manner in which weight changed as an animal underwent metamorphosis depended on the family and species it represented. For instance, in butterflies, such as the zebra longwing, gulf fritillary and monarch, weight change was minimal, but it was more pronounced in the giant swallowtail.

In contrast, the imperial moth, which pupates in an underground chamber, loses a remarkable 60% of its weight, and the cecropia moth, which constructs a double-layered cocoon in which it pupates, loses even more. Even within a single family or subfamily, weight change during metamorphosis may vary depending on a species’ biology: among the wooly bears used in the present study, the caterpillar of the acrea moth (also known as saltmarsh caterpillar) uses its own hairs to form its cocoon, and thus has much less need for silk, which is mostly used to bind these hairs together. Superficially similar-looking leopard moth caterpillar, on the other hand, spins the cocoon entirely from its own silk, adding droplets of bad-smelling repellent for chemical protection as they do so. I suspect this is the reason why a leopard moth caterpillar loses more weight during metamorphosis than a saltmarsh caterpillar. Intraspecifically, the weight can also vary substantially, as demonstrated by six leopard moth caterpillars in Figure 15.

## Data availability

### Underlying data

Raw data, including raw images and weights of caterpillars and pupa, are available on OSF, DOI: https://doi.org/10.17605/OSF.IO/D2P9H ( Sourakov, 2018c) and at https://doi.org/10.17605/OSF.IO/S5JXP ( Sourakov, 2020)

### Extended data

Figure S1. Full spectrum of heparin-induced wing pattern changes in dorsal hindwing of the common buckeye, *Junonia coenia* (A, B) Control group: (A) males and (B) females injected with H _2_O as prepupae. (C, D) Experimental group: (C) males and (D) females injected with heparin as prepupae. Wings are arranged left to right demonstrating a gradient in the reduction of the orange band (both groups) and expansion of the marginal bands (experimental group). DOI: https://doi.org/10.17605/OSF.IO/D2P9H ( Sourakov, 2018c).

Postscriptum - On the term Prepupa, including Figure S2: Morphological recognition of prepupa as a separate stage in Lepidoptera, with further subdivision into early prepupa (epp), prepupa (pp), and pharate pupa (php). (A-B) The common buckeye, *Junonia coenia*: (A) caterpillar; (B.i) early prepupa; (B.ii) prepupa; (C) The gulf fritillary, *Agraulis vanillae*: (C.i) early prepupa; (C.ii) prepupa; (C.iii) pharate pupa; (D) the giant swallowtail, *Heraclides cresphontes*: (D.i) caterpillar; (D.ii) early prepupa; (D.iii) prepupa. DOI: https://doi.org/10.17605/OSF.IO/D2P9H ( Sourakov, 2018c).

Table S1. Details concerning heparin injections for the specimens illustrated in Figures 1–7. DOI: https://doi.org/10.17605/OSF.IO/D2P9H ( Sourakov, 2018c).

Extended Specimen Data. Additional details concerning heparin injections for the specimens illustrated in Figures 1–13. DOI: [ https://doi.org/10.17605/OSF.IO/S5JXP] ( Sourakov (2020, October 30). F1000-Sourakov-V2-2020. https://doi.org/10.17605/OSF.IO/S5JXP).
