# Supplementary material for: Emperors, admirals and giants, zebras, tigers and woolly bears: casting a broader net in exploring heparin effects on Lepidoptera wing patterns
Source: F1000Res. 2020 Dec 4;7:1842. Originally published 2018 Nov 22. [Version 3] doi: 10.12688/f1000research.16926.3 (PMC7722536; doi:10.12688/f1000research.16926.3)
Supplement: Peer review history

# Peer review: Reviewer response for version 3

**DOI:** 10.5256/f1000research.30911.r75748

**Published:** 2020-12-07

**Authors:** Jeffrey M. Marcus

**Affiliations:** 1 Department of Biological Sciences, University of Manitoba, Winnipeg, MB, Canada

Thank you for updating the figure legend as I requested.

Is the work clearly and accurately presented and does it cite the current literature?

Partly

If applicable, is the statistical analysis and its interpretation appropriate?

Not applicable

Are all the source data underlying the results available to ensure full reproducibility?

Partly

Is the study design appropriate and is the work technically sound?

Yes

Are the conclusions drawn adequately supported by the results?

Partly

Are sufficient details of methods and analysis provided to allow replication by others?

Partly

Reviewer Expertise:

Evolution and development of butterfly color patterns, insect developmental genetics, and molecular phylogenetics.

I confirm that I have read this submission and believe that I have an appropriate level of expertise to confirm that it is of an acceptable scientific standard.

# Peer review: Reviewer response for version 2

**DOI:** 10.5256/f1000research.30488.r74594

**Published:** 2020-11-19

**Authors:** Arnaud Martin

**Affiliations:** 1 Department of Biological Sciences, The George Washington University, Washington, DC, USA

I enjoyed those many additions. 

 Notice the Fenner *et al*. (2020) work was conducted in the lab of Brian Counterman (not the Martin Lab) and is not relevant to eyespot; thus I would recommend editing:

*"For additional discussion of heparin influence pattern formation, see Sourakov & Shirai (2020)."*

Is the work clearly and accurately presented and does it cite the current literature?

Partly

If applicable, is the statistical analysis and its interpretation appropriate?

Not applicable

Are all the source data underlying the results available to ensure full reproducibility?

Yes

Is the study design appropriate and is the work technically sound?

Yes

Are the conclusions drawn adequately supported by the results?

Yes

Are sufficient details of methods and analysis provided to allow replication by others?

Partly

Reviewer Expertise:

Evolutionary Developmental Biology ; Lepidoptera

I confirm that I have read this submission and believe that I have an appropriate level of expertise to confirm that it is of an acceptable scientific standard.

# Peer review: Reviewer response for version 2

**DOI:** 10.5256/f1000research.30488.r74595

**Published:** 2020-11-12

**Authors:** Jeffrey M. Marcus

**Affiliations:** 1 Department of Biological Sciences, University of Manitoba, Winnipeg, MB, Canada

Thank you for addressing my earlier comments. I enjoyed reading about the new experiments in additional Lepidopteran species, especially in *Utetheisa*. In my review of version 2, I note that Figure 12, panel C lacks a description in the figure legend, without which it is not possible to evaluate the validity of the contents of the figure.

Is the work clearly and accurately presented and does it cite the current literature?

Partly

If applicable, is the statistical analysis and its interpretation appropriate?

Not applicable

Are all the source data underlying the results available to ensure full reproducibility?

Partly

Is the study design appropriate and is the work technically sound?

Yes

Are the conclusions drawn adequately supported by the results?

Partly

Are sufficient details of methods and analysis provided to allow replication by others?

Partly

Reviewer Expertise:

Evolution and development of butterfly color patterns, insect developmental genetics, and molecular phylogenetics.

I confirm that I have read this submission and believe that I have an appropriate level of expertise to confirm that it is of an acceptable scientific standard, however I have significant reservations, as outlined above.

# Response

**Published:** 2020-11-12

**Authors:** Andrei Sourakov

**Affiliations:** Florida Museum of Natural History, USA

Figure 12C illustrates a typical specimen from the control group.

# Peer review: Reviewer response for version 1

**DOI:** 10.5256/f1000research.18507.r41144

**Published:** 2018-12-21

**Authors:** Jeffrey M. Marcus

**Affiliations:** 1 Department of Biological Sciences, University of Manitoba, Winnipeg, MB, Canada

This manuscript expands on earlier work (including some by Sourakov, himself (Sourakov, 2017 ^1^) that uses heparin sodium sulfate salt to manipulate color pattern development in Lepidoptera. Most of the prior work had been done in a small number of species in the butterfly family Nymphalidae. This manuscript repeats experiments in the butterfly model system *Junonia coenia* (Nymphalidae) (Serfas and Carroll, 2005 ^2^) as controls and compares the results of these experiments to experiments using this technique in additional species of Nymphalid butterflies, as well as species of swallowtail butterflies (family Papilionidae) and of moth species in families Erebidae and Crambidae.

 While I enjoyed reading the manuscript, there are a number of changes that I would like to suggest to the author:

 Clarification of abstract:

1. In the methods section of the abstract, the species studied are only listed by their scientific names, while in the results section the species studied are only listed by common name.  Consequently, it would be challenging for anyone without an extensive knowledge of lepidopteran nomenclature to easily connect the experimental results with the appropriate species.
2. Suggest replacing the phrase “unusual eyespots” with the phrase “simple eyespots” in the results section of the abstract.

 Clarification of the introduction:

1. Adding heparin sodium sulfate does not alter the expression of Wnt-family ligands (= production of the Wnt proteins by cells), but instead alters their solubility, diffusion in the intercellural space, and activity (by altering their ability to bind to cell-surface receptors) (Fuerer *et al.* 2010 ^3^).
2. There is more than one Wnt ligand. As reported in (Martin and Reed, 2014 ^4^) and as discussed in (Martin and Courtier-Orgogozo, 2017 ^5^), there are at least 4 different Wnt ligands that are expressed in the developing butterfly wing during color pattern determination, with wingless (wg) and WntA perhaps being the most important. On the whole, it appears that heparin has a much greater effect on phenoytpes associated with WntA signaling than on phenotypes associated with wg signaling.

 Clarification of methods:

1. “Different concentrations and volumes of heparin sodium sulfate solution were injected.  Concentrations and volumes used were different for each species (according to their size), and were sometimes varied in order to achieve variation in response (Table S1)”. Please provide some sort of rationale for how dosage was adjusted. 
2. Also,Table S1 should include scientific names for each species and most importantly, the sample sizes of each and every trial listed in Table S1.

 Clarification of results and discussion:

1. “Ventrally the same individuals exhibited a reduction in the size of eyespots and the diffusion of some of the wing pattern elements.” Replace “diffusion” (which has clear and specific meaning in chemistry and physics that is not relevant here) with “loss of definition” or similar.
2. Referring to same sentence as R&D#1 immediately above as well as the paragraph that begins “The taxonomy of the genus *Junonia *in the New World…”. The effects of heparin treatment of color pattern strongly resemble some of the diagnostic phenotypes that distinguish the recently identified cryptic taxon *Junonia grisea* from *J. coenia *(including the reduction or elimination of orange parafocal elements on dorsal wing surfaces, reduction in size of dorsal forewing eyespots, and loss of definition of wing pattern elements on ventral wing surfaces (Lalonde and Marcus, 2018 ^6^). It should also be noted that different New World *Junonia* taxa, which cannot be distinguished by mitochondrial genotypes are distinguishable from one another by variation at the wingless locus (Borchers and Marcus, 2014 ^7^; Gemmell *et al.* 2014 ^8^; Peters and Marcus 2017 ^9^; Lalonde and Marcus, 2018 ^10^; Lalonde *et al.* 2018 ^6^).  It would be interesting indeed if these species-diagnostic color pattern phenotypes were actually caused by the evolution of Wnt ligand sequences and function.
3. “The normally present red spot in the center of the hindwing located on either side of M _3_…may become greatly reduced in heparin-injected individuals…” These red spots in *Heraclides cresphontes *are homologous to the WntA driven patterns in Nymphalid butterflies (see (Martin and Reed 2014 ^4^) and (Koch and Nijhout, 2002 ^11^).
4. I would like to see a greater synthesis of the findings of the present work with previous findings from heparin injections in *Automeris io* (Saturniidae) (Sourakov, 2017 ^1^), as well as some consideration of what these injections may actually be doing to the mechanism of Wnt signaling. Wnt ligands not only activate signal transduction though the canonical pathway (involving b-catenin), but also through alternative pathways: JNK, and Ca2+. The activation of different downstream pathways is one possible mechanism by which similar ligands could produce different color pattern outcomes, but which signalling pathways are triggered by each of the Wnt signals on lepidopteran wings is currently unknown (Özsu and Monteiro 2017 ^12^). 

I would like to encourage the author to consider these points in his revisions and also to continue his experimental explorations in future work.

Is the work clearly and accurately presented and does it cite the current literature?

Partly

If applicable, is the statistical analysis and its interpretation appropriate?

Not applicable

Are all the source data underlying the results available to ensure full reproducibility?

Partly

Is the study design appropriate and is the work technically sound?

Yes

Are the conclusions drawn adequately supported by the results?

Partly

Are sufficient details of methods and analysis provided to allow replication by others?

Partly

Reviewer Expertise:

Evolution and development of butterfly color patterns, insect developmental genetics, and molecular phylogenetics.

I confirm that I have read this submission and believe that I have an appropriate level of expertise to confirm that it is of an acceptable scientific standard, however I have significant reservations, as outlined above.

## References

[1] : Giving eyespots a shiner: Pharmacologic manipulation of the Io moth wing pattern. *F1000Res*.2017;6: 10.12688/f1000research.12258.2 1319 10.12688/f1000research.12258.2 29057069PMC5629545

[2] : Pharmacologic approaches to butterfly wing patterning: sulfated polysaccharides mimic or antagonize cold shock and alter the interpretation of gradients of positional information. *Dev Biol*.2005;287(2) : 10.1016/j.ydbio.2005.09.014 416-24 10.1016/j.ydbio.2005.09.014 16216238

[3] : Lentiviral vectors to probe and manipulate the Wnt signaling pathway. *PLoS One*.2010;5(2) : 10.1371/journal.pone.0009370 e9370 10.1371/journal.pone.0009370 20186325PMC2826402

[4] : Wnt signaling underlies evolution and development of the butterfly wing pattern symmetry systems. *Dev Biol*.2014;395(2) : 10.1016/j.ydbio.2014.08.031 367-78 10.1016/j.ydbio.2014.08.031 25196151

[5] : Predicting the genetic loci of past evolution. *bioRxiv*.2017; 10.1101/205153 10.1101/205153

[6] : The Taxonomy and Population Structure of the Buckeye Butterflies (GenusJunonia , Nymphalidae: Nymphalini) of Florida, USA. *Journal of the Lepidopterists' Society*.2018;72(2) : 10.18473/lepi.v72i2.a2 97-115 10.18473/lepi.v72i2.a2

[7] : Genetic population structure of buckeye butterflies (Junonia ) from Argentina. *Systematic Entomology*.2014;39(2) : 10.1111/syen.12053 242-255 10.1111/syen.12053

[8] : Molecular Population Structure ofJunonia Butterflies from French Guiana, Guadeloupe, and Martinique. *Psyche: A Journal of Entomology*.2014;2014: 10.1155/2014/897596 1-21 10.1155/2014/897596

[9] : Taxonomy as a hypothesis: testing the status of the Bermuda buckeye butterflyJunonia coenia bergi (Lepidoptera: Nymphalidae). *Systematic Entomology*.2017;42(1) : 10.1111/syen.12214 288-300 10.1111/syen.12214

[10] : Getting western: biogeographical analysis of morphological variation, mitochondrial haplotypes and nuclear markers reveals cryptic species and hybrid zones in theJunonia butterflies of the American southwest and Mexico. *Systematic Entomology*.2018; 10.1111/syen.12335 10.1111/syen.12335

[11] : The role of wing veins in colour pattern development in the butterfly Papilio xuthus (Lepidoptera: Papilionidae). *European Journal of Entomology*.2002;99(1) : 10.14411/eje.2002.012 67-72 10.14411/eje.2002.012

[12] : Wound healing, calcium signaling, and other novel pathways are associated with the formation of butterfly eyespots. *BMC Genomics*.2017;18(1) : 10.1186/s12864-017-4175-7 788 10.1186/s12864-017-4175-7 29037153PMC5644175

# Peer review: Reviewer response for version 1

**DOI:** 10.5256/f1000research.18507.r41054

**Published:** 2018-12-13

**Authors:** Arnaud Martin

**Affiliations:** 1 Department of Biological Sciences, The George Washington University, Washington, DC, USA

Introduction:

- "Altering the expression of a gene ligand, like Wnt, through injecting heparin at the crucial stages of wing pattern development, can provide a glimpse into the mechanisms underlying wing pattern evolution and development". Replace "expression of a gene ligand" by "activity of a signaling ligand". Binari et al ^1^ could be cited here.
- "Eurytides marcellus", replace with "Protographium" (as well as elsewhere in the manuscript)

Methods:

- Provide the Molecular Weight of the commercial source of Heparin sodium sulfate in kDa (shall be indicated on the container)

Table S1:

- Please convert all the dosage estimates into ng. For instance, 1uL of 5% = 5ug/100nL corresponds to a 50ug dose. This allows a more direct comparison of all the experiments.
- What were the conditions used on the species that did not provide meaningful results?

Results:

- Acrea / Leopard moths. Since heparin tends to mimic the effect of cold-shocks, it would be expected to find natural aberrants of those species that look similar to the heparin treatment. By any chance, are there MGCL specimens that go along similar axes of variation?

Asterocampa:

 Regarding: "Marginal eyespots in many Nymphalidae are concentrically organized and serve as models for studies of development, as for example, in Bicyclus anynana, where they have been shown to be positively regulated by Wingless (Wnt) (Özsu et al., 2017 ^2^). Wnt signaling delimits the boundaries of wing spots, as reviewed by Martin & Courtier-Orgogozo.,2017 ^3^, and is affected by heparin. Figure 4(Aiii–Diii) suggests that, even though the serial border spots of the tawny emperor are not as concentrically organized as in many other nymphalids, they are nevertheless homologous. The marginal bands migrating basally under the influence of heparin are inhibited from invading eyespot centers."

 It shall be noted 1) that WntA Knock-Outs results in marginal effects in various nymphalids (Mazo-Vargas et al.,2017 ^4^), shifting the distal Parafocal Elements towards the margin, leaving the hindwing eyespots unaffected, but reducing the size of the WntA-positive forewing eyespots and 2) that the current results in Asterocampa hindwings (and perhaps forewings) seem analagous to the effects of Heparin in Vanessa cardui, as reported in Martin and Reed Dev. Biol. 2014 ^5^.

 To me, the Asterocampa heparin results are best explained by analogy with the Vanessa system (also analyzed by Otaki in several articles such as Ataki et al.,2008 ^6^. Asterocampa shows a similar color inversion effect on hindwing eyespots (both ventral and dorsal) to Vanessa heparin injected specimens. This must be independent of WntA, because WntA KOs do not affect the Vanessa hindwing, and the effect of heparin on those Asterocampa/Vanessa hindwing eyespots could be explained by other morphogens, possibly an eyespot focal Wg signal as proposed in Bicyclus (Özsu et al., 2017 ^2^), non-Wnt heparin-sensitive morphogens, or a complex interaction dynamics between wing margin signals and eyespot color patterning (the Nijhout book, or some Otaki references goes into classic surgery experiments). Notice however that one could expect an expansion of eyespots upon heparin injection if a focal eyespot Wg signal was in place. Oliver et al.,2012 ^7^ provide comparative and developmental evidence of complex interactions between the margin and the eyespot positioning process, so I think that overall we are still scratching the surface of the iceberg and the effects of heparin in that area are difficult to interpret. 

 Discussion:

- Junonia: provide more comparisons of the results with those reported by Serfas and Carroll. It seems to me that the pre-pupal injection results in less striking effects than the early pupal ones, for instance on the Wg-positive Discalis elements (orange stripes in Junonia forewings)?
- Weight loss data: please provide more data on the phenomenon of fluid excretion (time, amount?) by the silk-spinning species.

Is the work clearly and accurately presented and does it cite the current literature?

Partly

If applicable, is the statistical analysis and its interpretation appropriate?

Not applicable

Are all the source data underlying the results available to ensure full reproducibility?

Yes

Is the study design appropriate and is the work technically sound?

Yes

Are the conclusions drawn adequately supported by the results?

Yes

Are sufficient details of methods and analysis provided to allow replication by others?

Partly

Reviewer Expertise:

Evolutionary Developmental Biology ; Lepidoptera

I confirm that I have read this submission and believe that I have an appropriate level of expertise to confirm that it is of an acceptable scientific standard, however I have significant reservations, as outlined above.

## References

[1] : Genetic evidence that heparin-like glycosaminoglycans are involved in wingless signaling. *Development*.1997;124(13) :2623-32 921700410.1242/dev.124.13.2623

[2] : Wound healing, calcium signaling, and other novel pathways are associated with the formation of butterfly eyespots. *BMC Genomics*.2017;18(1) : 10.1186/s12864-017-4175-7 788 10.1186/s12864-017-4175-7 29037153PMC5644175

[3] : Predicting the genetic loci of past evolution. *bioRxiv*.2017; 10.1101/205153 10.1101/205153

[4] : Macroevolutionary shifts ofWntA function potentiate butterfly wing-pattern diversity. *Proceedings of the National Academy of Sciences*.2017;114(40) : 10.1073/pnas.1708149114 10701-10706 10.1073/pnas.1708149114 PMC563589428923954

[5] : Wnt signaling underlies evolution and development of the butterfly wing pattern symmetry systems. *Dev Biol*.2014;395(2) : 10.1016/j.ydbio.2014.08.031 367-78 10.1016/j.ydbio.2014.08.031 25196151

[6] : Physiologically induced color-pattern changes in butterfly wings: mechanistic and evolutionary implications. *J Insect Physiol*.2008;54(7) : 10.1016/j.jinsphys.2008.05.006 1099-112 10.1016/j.jinsphys.2008.05.006 18638480

[7] : A single origin for nymphalid butterfly eyespots followed by widespread loss of associated gene expression. *PLoS Genet*.2012;8(8) : 10.1371/journal.pgen.1002893 e1002893 10.1371/journal.pgen.1002893 22916033PMC3420954
